# Butyrate modifies intestinal barrier function in IPEC-J2 cells through a selective upregulation of tight junction proteins and activation of the Akt signaling pathway

**DOI:** 10.1371/journal.pone.0179586

**Published:** 2017-06-27

**Authors:** Hui Yan, Kolapo M. Ajuwon

**Affiliations:** Department of Animal Sciences, Purdue University, West Lafayette, Indiana, United States of America; Emory University School of Medicine, UNITED STATES

## Abstract

The intestinal epithelial barrier, composed of epithelial cells, tight junction proteins and intestinal secretions, prevents passage of luminal substances and antigens through the paracellular space. Dysfunction of the intestinal barrier integrity induced by toxins and pathogens is associated with a variety of gastrointestinal disorders and diseases. Although butyrate is known to enhance intestinal health, its role in the protection of intestinal barrier function is poorly characterized. Therefore, we investigated the effect of butyrate on intestinal epithelial integrity and tight junction permeability in a model of LPS-induced inflammation in IPEC-J2 cells. Butyrate dose-dependently reduced LPS impairment of intestinal barrier integrity and tight junction permeability, measured by trans-epithelial electrical resistance (TEER) and paracellular uptake of fluorescein isothiocyanate-dextran (FITC-dextran). Additionally, butyrate increased both mRNA expression and protein abundance of claudins-3 and 4, and influenced intracellular ATP concentration in a dose-dependent manner. Furthermore, butyrate prevented the downregulation of Akt and 4E-BP1 phosphorylation by LPS, indicating that butyrate might enhance tight junction protein abundance through mechanisms that included activation of Akt/mTOR mediated protein synthesis. The regulation of AMPK activity and intracellular ATP level by butyrate indicates that butyrate might regulate energy status of the cell, perhaps by serving as a nutrient substrate for ATP synthesis, to support intestinal epithelial barrier tight junction protein abundance. Our findings suggest that butyrate might protect epithelial cells from LPS-induced impairment of barrier integrity through an increase in the synthesis of tight junction proteins, and perhaps regulation of energy homeostasis.

## Introduction

The gastrointestinal epithelium is the largest exchange surface between the host and the external environment [1]. It is composed of a monolayer of intestinal epithelial cells that provide a physical barrier. The intestinal epithelial barrier allows absorption of nutrients in the diet and prevents passage of pathogens and toxins into systemic circulation. Abundant evidence indicate that an intact and healthy intestinal barrier is necessary for optimal health [2]. Defective intestinal epithelial barrier, characterized by increased intestinal permeability is positively correlated with a variety of gastrointestinal dysfunctions and diseases [2]. In pigs, pathogenic enteric bacteria, mycotoxin, and various stress inducing factors, such as weaning and heat stress, are potent disruptors of intestinal barrier function, leading to impaired growth and digestive disorders, diarrhea and other gastrointestinal discomfort [3–7]. In poultry, *Salmonella* infection, toxins, and heat stress induce an increase in intestinal epithelial permeability, resulting in nutrient malabsorption, mortality and potential human foodborne salmonellosis [8–10]. In humans, impaired intestinal barrier function is associated with a wide range of diseases, such as inflammatory bowel disease [11], necrotizing enterocolitis [12], type I diabetes mellitus [13], and rheumatic diseases [14]. The intestinal epithelial barrier is mainly sustained by tight junction proteins, which are apical multi-protein complexes [15]. Tight junctions hold adjacent epithelial cells at the apical side of the lateral membrane and anchor transmembrane proteins (claudins and occludin) to intracellular actin cytoskeleton [16]. They play a crucial role in the regulation of paracellular permeability and maintenance of epithelium integrity [15–17]. Therefore, intestinal tight junctions are considered as therapeutic target for the modulation of intestinal barrier function and the prevention of various gastrointestinal diseases.

Short chain fatty acids (SCFA) are products of microbial fermentation of indigestible carbohydrates in the large intestine as we previously showed [18]. In this study [18], we determined that cecal butyrate concentration was lower in pigs fed a high fat diet compared to those on a low-fat diet. Obesity and consumption of a high fat diet is known to cause impaired gut barrier integrity indicated by the higher incidence of leaky gut on obesity [19, 20], and this may be related to reduced butyrate production in obese subjects. This is because recent studies confirm that SCFA, especially butyrate, may play an important role in the maintenance of intestinal barrier function. In the gastrointestinal tract (GIT), butyrate is preferentially taken up by colonic epithelial cells for use as energy source, which in turn promotes epithelial cell proliferation and injury repair [15, 21]. Butyrate in particular has been shown to prevent colonization of enteric pathogens in the GIT through the upregulation of expression of the epithelial antimicrobial peptide in rabbits [22], and the modulation of virulence gene expression in colonocytes [23]. Butyrate also decreases intestinal permeability and enhances assembly of tight junctions in Caco-2 cell model [24, 25]. However, there is a dearth of information on the mechanism of butyrate effect on the regulation of tight junction integrity in the small intestinal epithelium, which plays a key role in immune response and nutrient absorption.

In this study, we used the porcine intestinal epithelial cell line (IPEC-J2), originally from the jejunum of a neonatal piglet [26], as the experimental model to investigate the mechanism of butyrate effect in the gut, because these cells are highly sensitive to lipopolysaccharide (LPS) stimulation leading to induction of inflammation and the impairment of intestinal epithelial integrity [27, 28], unlike CACO-2 cells which are hyporesponsive [29]. LPS challenge induces defective or leaky tight junctions through binding to toll like receptor-4 (TLR-4) and activation of NFκB signaling [30, 31]. Inflammatory cytokines, produced as a result of NFκB activation, can lead to break down of the tight junction [32, 33] and cause inhibition of Akt/mTOR mediated protein synthesis [34–36]. Therefore, the aim of the present study was to determine, in the IPEC-J2 model, the mechanism of the opposing effect of butyrate on intestinal epithelial integrity and tight junction disruption induced by LPS. Herein, we report that butyrate mitigated the negative effect of LPS on epithelial integrity, concurrent with a selective upregulation of expression of tight junction proteins and an increase in intracellular ATP level.

## Materials and methods

### Cell culture conditions

The Intestinal porcine epithelial cell (IPEC-J2) model was a kind gift from Dr. Nicholas Gabler (Iowa State University, Ames, IA). Cells were cultured in a humidified incubator at 37°C under 5% CO_2_ in 25 cm^2^ cell culture flask (Corning Inc., Corning, NY). Cells were grown in Dulbecco’s modified Eagle’s medium: Nutrient Mixture F-12 (DMEM/F12; Sigma-Aldrich, St. Louis, MO) with 5% fetal bovine serum (FBS; Mediatech. Inc., Manassas, VA) supplemented with 1% insulin-transferrin-selenium premix (ITS premix: human recombinant insulin 1 mg/ml, transferrin 0.6 mg/ml and selenium 0.6 μg/ml; Corning Inc., Corning, NY), 5 ng/ml epidermal growth factor (EGF: Sigma-Aldrich, St. Louis, MO), and 1% penicillin-streptomycin mixture (Mediatech. Inc., Manassas, VA). Cells were seeded into 24-well cell culture plates (BD Falcon, Corning Inc., Corning, NY) at 0.5 × 10^5^ cells/ml to form a confluent monolayer within 4 days, and then switched to the same medium without FBS to induce differentiation. Medium was replaced every 2 days, and cells were challenged with LPS on day 7 post-differentiation.

### Butyrate treatment and LPS challenge

Sodium butyrate (Sigma-Aldrich, St. Louis, MO) was dissolved in DMEM/F12 to make the butyrate working solution. Cells were treated with butyrate throughout the differentiation period (day 0 to 7). On day 7 post-differentiation, fresh medium was added and cells were challenged with LPS-derived from *E*. *coli* O55:B5 (Sigma-Aldrich, St. Louis, MO) at 10 μg/ml. Preliminary experiments were also conducted to determine the optimal butyrate concentration and duration of exposure to LPS to determine effect on cell viability. Analysis of cell viability was performed in 96-well cell culture plates (Corning Inc., Corning, NY) with the use of CellQuanti-Blue^TM^ cell viability assay kit (BioAssay Systems, Hayward, CA), according to the manufacturer’s instruction. The results of pilot experiments indicated that optimal concentrations of butyrate ranged from 0.1 mM to 1 mM because there was no significant adverse effect on cell viability at these concentrations (S1 Fig). Stimulation with LPS at 10 μg/ml LPS did not cause any cytotoxic effects up to 24 h of culture. There were 4 to 6 replicates per experiment.

### RNA extraction and real-time PCR analysis

Total cellular RNA was isolated at the end of LPS treatment using TRIzol® reagent (Invitrogen, Carlsbad, CA). RNA concentration and purity were determined by spectrophotometry using the Nanodrop ND-1000 spectrophotometer (Thermo Fisher Scientific Inc. Waltham, MA). Reverse transcription was carried out with 1 μg RNA using MMLV reverse transcriptase (Promega, Madison, WI) to generate the cDNA. Quantitative real-time PCR was performed on the MyiQ real-time PCR detection system (Bio-Rad, Hercules, CA) using the SYBR green 2× RT-PCR mix (Qiagen, Foster City, CA). The mRNA expression of the following genes was determined: inflammation-related genes; TNFα (tumor necrosis factor alpha), MCP-1 (monocyte chemoattractant protein), IL (interleukin) -8, IL-1β, IL-10 and TLR-4, and tight junction proteins: claudin-1, claudin-3, claudin-4, occludin and zonula occludens (ZO)-1. Abundance of specific mRNA transcripts was normalized to 18S ribosomal RNA using Pfaffl’s method [37]. Primers for PCR amplification were designed based on the NCBI database and listed in Table 1.

**Table 1:** List of PCR primers.

**Gene**	**Forward**	**Reverse**
GAPDH	5'-GGG CAT GAA CCA TGA GAA GT-3'	5'-TGT GGT CAT GAG TCC TTC CA-3'
TNFα	5'-CGT CGC CCA CGT TGT AGC CAA T-3'	5'-GCC CAT CTG TCG GCA CCA CC-3'
IL-6	5'-TCT GGG TTC AAT CAG GAG ACC TGC-3'	5'-TGC ACG GCC TCG ACA TTT CCC-3'
IL-8	5'-TTT CTG CAG CTC TCT GTG AGG-3’	5'-CTG CTG TTG TTG TTG CTT CTC-3’
TLR4	5'-ATC TGA GAG CTG GGA CCC TT-3’	5'-ATG TGG GGA TGT TGT CAG GG-3’
MCP-1	5'-CAC CAG CAG CAA GTG TCC TA-3’	5'-TCC AGG TGG CTT ATG GAG TC-3’
IL-1b	5'-CCA AAG AGG GAC ATG GAG AA-3’	5'-GGG CTT TTG TTC TGC TTG AG-3’
Claudin-1	5'-TTT CCT CAA TAC AGG AGG GAA GC-3'	5'-CCC TCT CCC CAC ATT CGA G-3'
Claudin-3	5'-GCC AAG ATC CTC TAC TCC GC-3'	5'-GAG AGC TGC CTA GCA TCT GG-3'
Claudin-4	5'-CTC TCG GAC ACC TTC CCA AG-3'	5'-GCA GTG GGA AGG TCA AAG G-3'
Occludin	5'-CTA CTC GTC CAA CGG GAA AG-3'	5'-ACG CCT CCA AGT TAC CAC TG-3'
ZO-1	5'-AAG CCC TAA GTT CAA TCA CAA TCT-3'	5'-ATC AAA CTC AGG AGG CGG C-3'

Primers were designed with the NCBI Primer Blast program with sequences in the NCBI database.

### Epithelial cell integrity determination

Epithelial cell integrity was assessed through the measurement of trans-epithelial electrical resistance (TEER) [38] and paracellular permeability in a 12-well trans-well plate (Corning Inc., Corning, NY). IPEC-J2 cells were seeded into 1.12 cm^2^ Transwell-COL inserts with 0.4 μm pore membrane (Corning Inc., Corning, NY) at a density of 1 × 10^5^ cells/ml. Confluent cells were induced to differentiate as stated above with butyrate supplementation on both the apical and basolateral sides of the cell monolayer. On day 9 post-differentiation, cells were treated with 10 μg/ml LPS on the apical side to mimic bacterial contact with intestinal epithelial cells in the lumen, and TEER was measured at 0, 12, 24 h, respectively. TEER measurements were conducted using a Millicell ERS-2 Voltohmmeter® (Millipore, Billerica, MA) and the values were expressed as Ohm × cm^2^ (Ωcm^2^). Although LPS may also activate toll-like receptor 2 (TLR2), [39]we focused mainly on TLR4 because it is recognized as the major receptor for LPS [40].

After 24 h LPS stimulation, paracellular permeability was measured using 4-kDa fluorescein isothiocyanate-dextran (FITC-dextran; Sigma-Aldrich, St. Louis, MO). Dextran was dissolved in DMEM/F12 medium and loaded on apical side of transwell insert at a concentration of 2.2 g/L [41]. After 2 h of incubation, the amount of fluorescence on the basolateral side was measured using fluorometry (Tecan Group Ltd., Switzerland) with an excitation and emission wavelengths of 490 nm and 520 nm, respectively.

### Measurement of secreted cytokine concentrations by ELISA

After 8 h LPS challenge, cell culture media was collected and centrifuged at 12,000 g for 10 min at 4°C for analysis cytokine concentration by ELISA. Levels of secreted inflammatory cytokines, IL-8, IL-10 and TNFα, were measured in the culture media using solid phase ELISA (enzyme-linked immune-sorbent assay) with commercially available kits (Porcine CXCL8/IL-8 Quantikine ELISA kit, Porcine IL-10 ELISA kit, and Porcine TNFα Quantikine ELISA kit; R&D system Inc., Minneapolis, MN), according to the manufacturer’s instructions.

### Immunoblotting analyses

Cells were rinsed with 1× phosphate buffered saline (PBS) and scraped in 1× RIPA lysis buffer (50 mM Tris-HCl, 15mM NaCl, 10 mM EDTA, 0.25% deoxycholic acid, 0.1% Triton X, 0.01% Na_3_VO_4_) with a mixture of phosphatase and protease inhibitors (Sigma-Aldrich, St. Louis, MO). Cell lysates were centrifuged at 10,000 g for 15 min at 4°C. Supernatants were collected for immunoblotting analysis. Protein concentrations were determined the using the bicinchoninic acid (BCA) assay kit (Sigma-Aldrich, St. Louis, MO). Equal amounts of proteins were resolved through SDS-PAGE (sodium dodecyl sulfate polyacrylamide gel electrophoresis) on 7.5% -12.5% polyacrylamide gels. Proteins were then transferred onto 0.2 μm nitrocellulose membranes (Bio-Rad, Hercules, CA) with a semi-dry trans-membrane transfer system (Bio-Rad, Hercules, CA). The membranes were blocked with 5% bovine serum albumin (BSA) in Tris-buffered saline with 0.1% Tween (TBST: 50mM Tris-HCl, 150mM NaCl, 0.1% Tween 20, pH 7.4), and incubated in specific primary antibodies at 4°C overnight. The following primary antibodies were used: Claudin-1, Claudin-3, Claudin-4, Occludin, ZO-1 (Zonula occludens) (Invitrogen, Carlsbad, CA), Akt, phospho-Akt, AMPKα (AMP-activated protein kinase), phospho-AMPKα, 4EBP1 (eukaryotic translation initiation factor 4E-binding protein 1), phospho-4E-BP1, IκB, and β-actin (Cell Signaling Technology, Danvers, MA). Membranes were then incubated with secondary antibodies (anti-rabbit or anti-mouse IgG-HRP, horseradish peroxidase conjugated anti-rabbit or anti-mouse IgG) (Cell Signaling Technology, Danvers, MA) at a dilution of 1: 20,000. Visualization was performed using the Immobilon® chemiluminescent HRP substrate (Millipore, Billerica, MA) on an autoradiographic film (Santa Cruz Biotechnology, Santa Cruz, CA). Signal densities of immunoblotting image were determined using a Kodak EDAS290 image system (Kodak, New Haven, CT) [42]. Original images of western blots are presented in supporting information file S2 Fig.

### Intracellular ATP determination

Cell lysates were collected 8 h after LPS challenge using 1× RIPA lysis buffer and centrifuged to obtain cell supernatant as described above. Intracellular ATP was measured in the supernatant using an ATP Determination Kit (Invitrogen, Carlsbad, CA) according to the manufacturer’s protocols. ATP values were then normalized to protein concentrations determined with the BCA assay kit (Sigma-Aldrich, St. Louis, MO).

### Statistical analyses

Data were analyzed through one-way analysis of variance (ANOVA) using the GLM procedure of SAS (SAS Inst. Inc., Cary, NC). Tukey multiple comparison analysis was performed to determine significance of differences among treatment means. Results were expressed as mean ± SE. Significant difference was set at P-value less than 0.05, whereas a P-value between 0.05 and 0.1 was considered a tendency. Summarized data points used for the analyses are presented in supporting information file S1 Text.

## Results

### Butyrate increases epithelial cell integrity against LPS-induced damage

**Fig 1:**
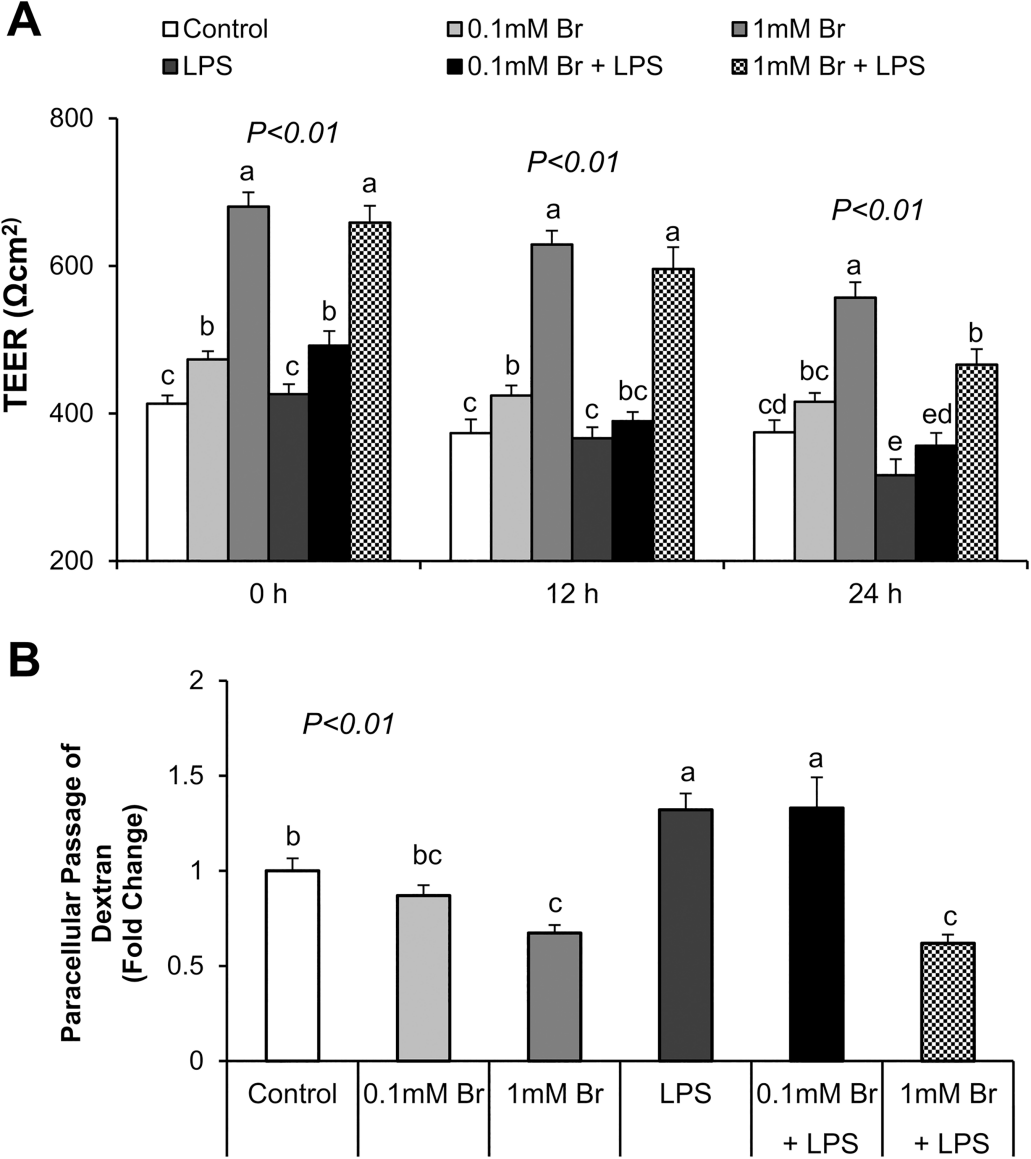
Effects of butyrate and LPS on intestinal barrier integrity measured by TEER and paracellular permeability in IPEC-J2 cells. (A) Cells were challenged with LPS on day 9 post-differentiation; TEER was measured at 0, 12, and 24 h after LPS challenge, respectively. (B) Paracellular permeability was determined by measuring FITC-dextran flux at 24 h after LPS challenge. Data were analyzed by one-way ANOVA with Tukey multiple comparison test. Values are means ± SE, n = 4. Different superscript letters on bars (a, b, c, d, e) indicate significant mean differences, P < 0.05.

We investigated the effect of butyrate and LPS on epithelial cell integrity by measuring TEER at 0, 12 and 24 h of LPS stimulation and paracellular dextran passage 24 h post-challenge. We observed that LPS exposure significantly decreased TEER (P<0.05) at 24 h (Fig 1A) and increased passage of FITC-dextran (P<0.05) (Fig 1B), indicating that LPS impaired epithelial barrier integrity. Pretreatment of cells with butyrate during differentiation significantly increased TEER (P<0.05) and decreased the paracellular flux of FITC-dextran (P<0.05) in a dose-dependent manner (Fig 1). Specifically, treatment with butyrate at the 1 mM concentration prevented LPS induced increases in paracellular permeability as shown by the reduced passage of FITC-dextran (Fig 1B).

These results provided initial evidence indicated that butyrate could protect cells from LPS-induced increase in paracellular permeability and damage to epithelial barrier integrity.

### Butyrate increases expression of tight junction claudins against LPS-induced reduction

**Fig 2:**
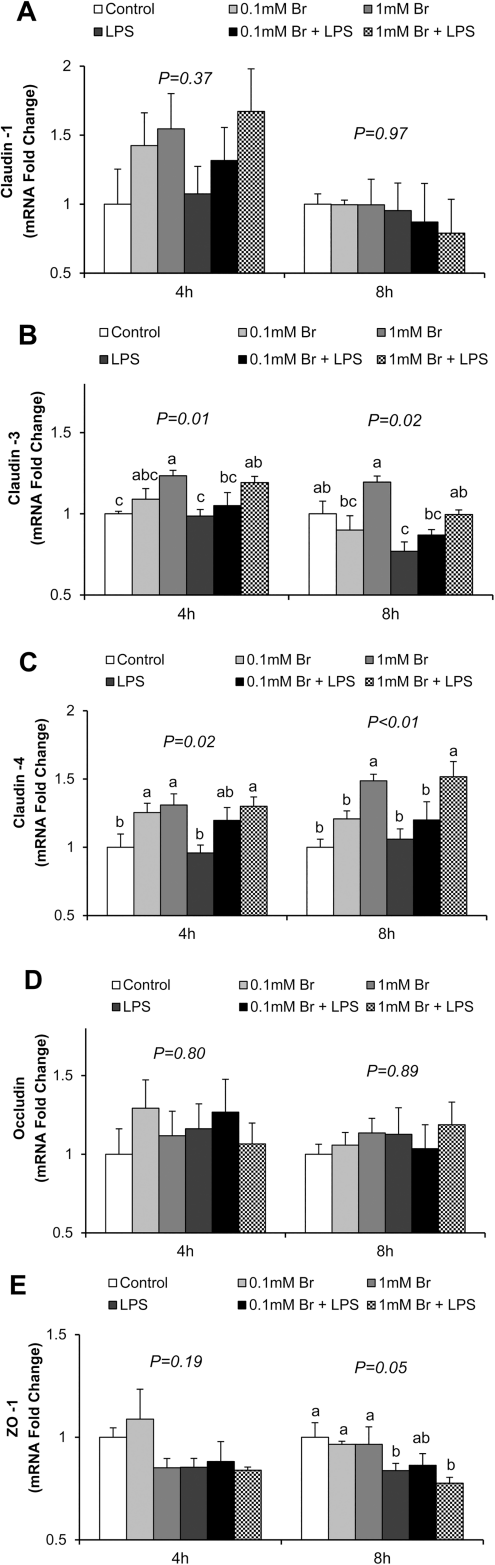
Effect of butyrate and LPS on mRNA expression of tight junction proteins in IPEC-J2 cells. Real time PCR was performed to determine mRNA expression of (A) claudin-1, (B) claudin-3, (C) claudin-4, (D) occludin and (E) ZO-1 at 4 and 8 h after LPS challenge, respectively. Data were analyzed by one-way ANOVA with Tukey multiple comparison test. Values are means ± SE, n = 6. Different superscript letters on bars (a, b, c) indicate significant differences, P < 0.05.

**Fig 3:**
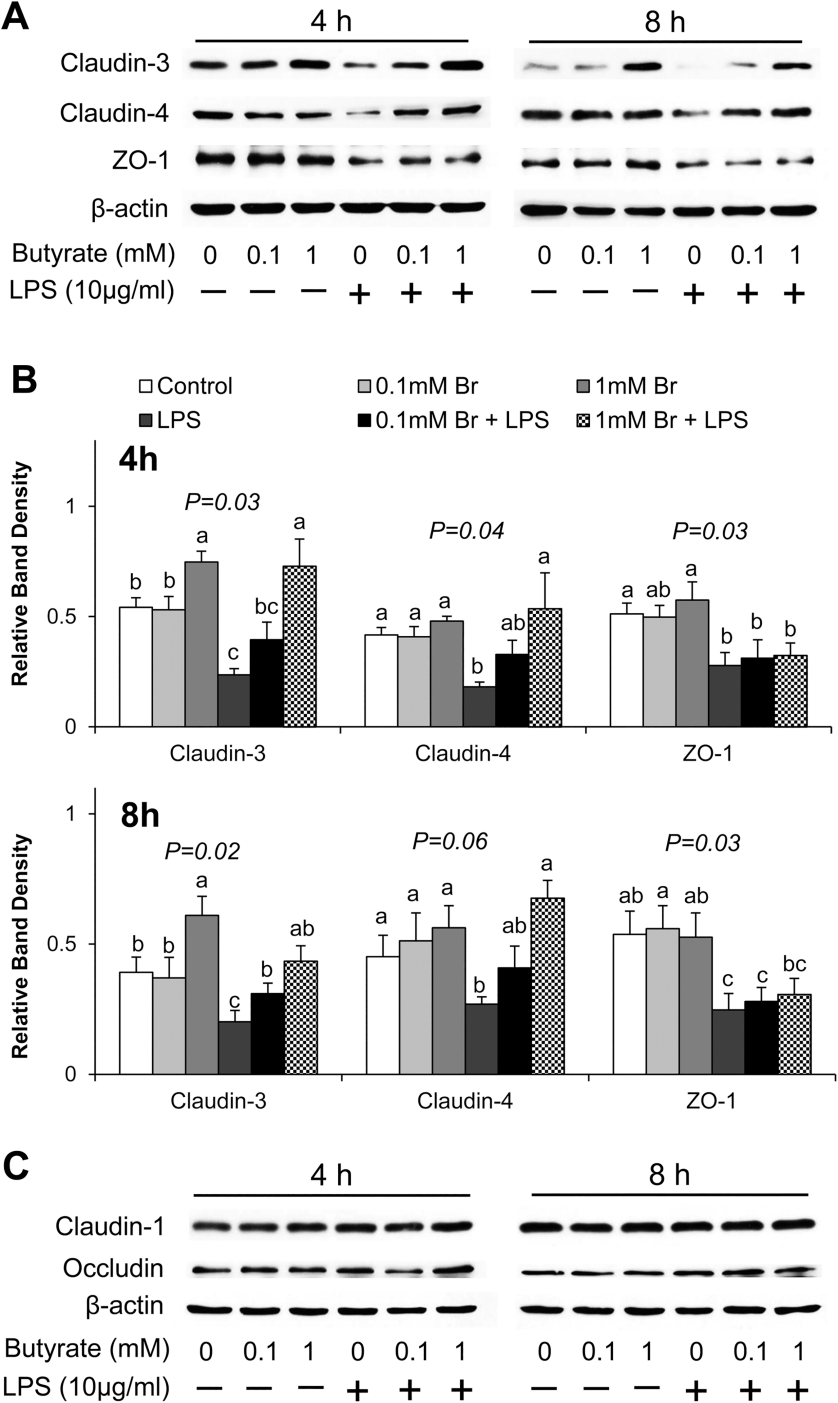
Effect of butyrate and LPS on abundance of tight junction proteins in IPEC-J2 cells. Cell lysates were isolated after 4 and 8 h of LPS challenge, and subjected to immunoblotting analysis. Data were analyzed by one-way ANOVA with Tukey multiple comparison test. Values are means ± SE, n = 4. Different superscript letters (a, b, c) on bars indicate significant mean differences, P < 0.05.

Therefore, we further investigated whether butyrate exerted a protective effect by regulating expression of tight junction proteins. Using real-time PCR (Fig 2) and immunoblotting (Fig 3), we determined that LPS exposure decreased claudin-3 mRNA expression (P<0.05) after 8 h of stimulation (Fig 2B) and decreased both claudin-3 and claudin-4 protein levels (P<0.05) after 4 h and 8 h of stimulation (Fig 3). Meanwhile, pretreatment of cells with butyrate, especially at the 1 mM concentration, significantly increased both the mRNA and protein expression of claudin-3 and claudin-4, and prevented the LPS-induced decreases in these proteins (Figs 2 and 3A). LPS treatment also led to decreases in mRNA (Fig 2E) and protein (Fig 3) abundance of ZO-1; however, no effect of butyrate was observed. Expression of claudin-1 and occludin was also investigated, but no significant influences of both LPS and butyrate were detected (Figs 2 and 3). These results demonstrate that butyrate has a mitigating effect on the downregulation of expression of claudin-3 and claudin-4 induced by LPS.

### Butyrate induces inflammatory cytokine expression in intestinal epithelial cells

**Fig 4:**
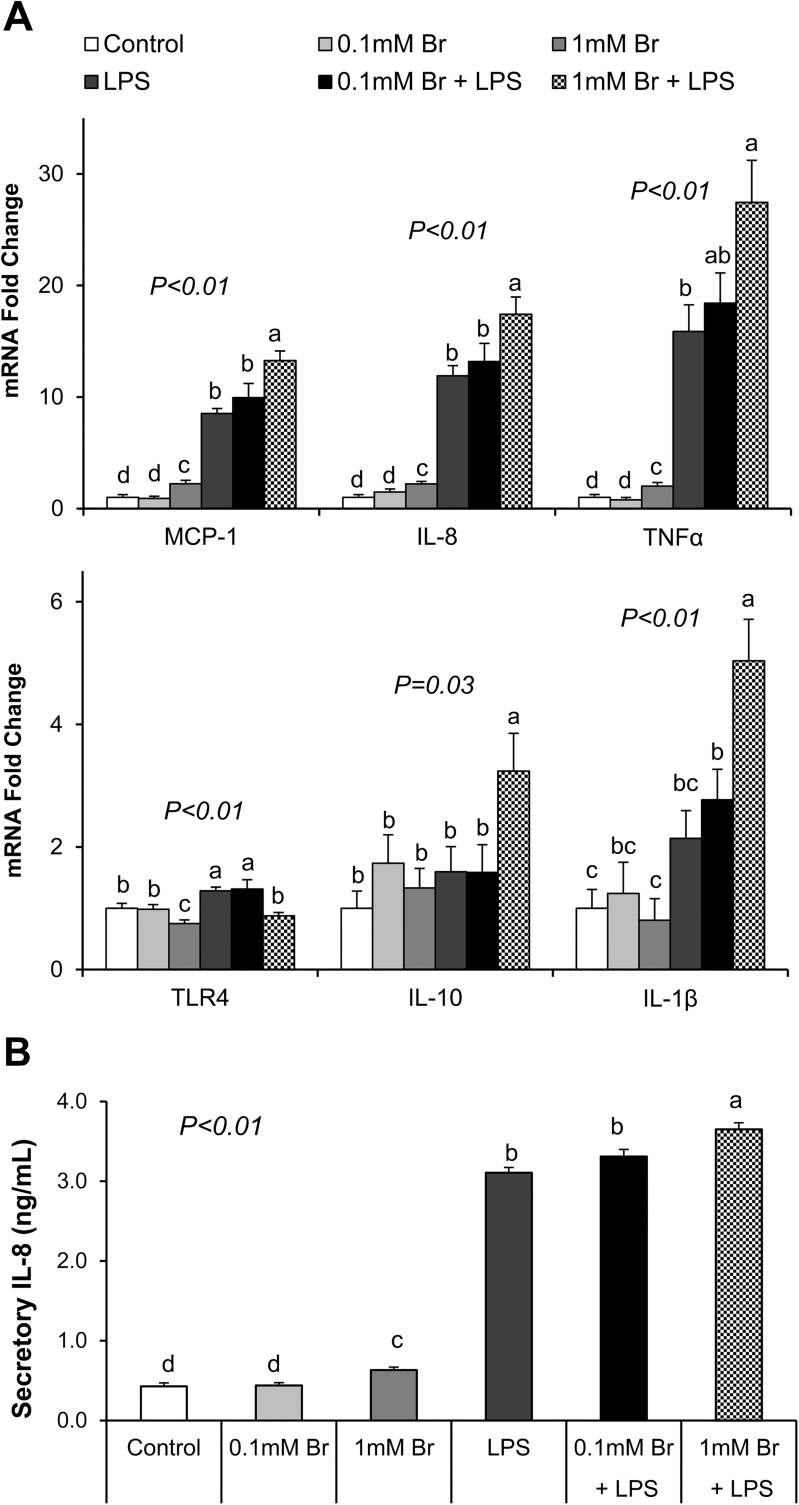
Effect of butyrate and LPS on mRNA expression of inflammatory related genes and secretion of IL-8 in IPEC-J2 cells. (A) mRNA expression of inflammation related genes after 8 h LPS challenge. (B) Level of secretory IL-8 in cell culture media after 8 h of LPS. Data were analyzed by one-way ANOVA with Tukey multiple comparison test. Values are means ± SE, n = 6. Different superscript letters (a, b, c, d) on bars indicate significant mean differences, P < 0.05.

We found that LPS stimulation induced mRNA expression of inflammatory cytokines (MCP-1, IL-8, TNFα) as well as IL-8 protein secretion (P<0.05) (Fig 4). Interestingly, butyrate at the 1mM concentration also mildly, albeit significantly, induced the expression of TNFα, IL-8 and MCP-1 compared to the control, and this effect was independent of LPS exposure (P<0.05) (Fig 4). In addition, butyrate at the 1mM concentration also enhanced IL-10 and IL-1β expression, but only in LPS-treated cells (P<0.05) (Fig 4A). Levels of IL-10 and TNFα proteins secreted into the medium were also investigated, but their levels were too low to be detected.

### Butyrate downregulates TLR-4 expression

It is known that LPS induces inflammatory cytokine expression through binding and activation of TLR-4, leading to activation of the NFκB signaling. In this study, LPS exposure significantly increased mRNA expression of TLR-4 after 4 h (P<0.01), whereas pretreatment of cells with butyrate inhibited its induction (P<0.01) (Fig 4A). This suggests that butyrate might block LPS-induced increases in TLR-4 expression, perhaps to limit TLR-4 mediated signaling. However, lack of a good antibody prevented us from measuring TLR-4 protein abundance. Thus, it is still unclear if LPS leads to a reduction in TLR-4 protein expression in these cells.

### Butyrate activates Akt/mTOR signaling and opposes LPS effect on this pathway

**Fig 5:**
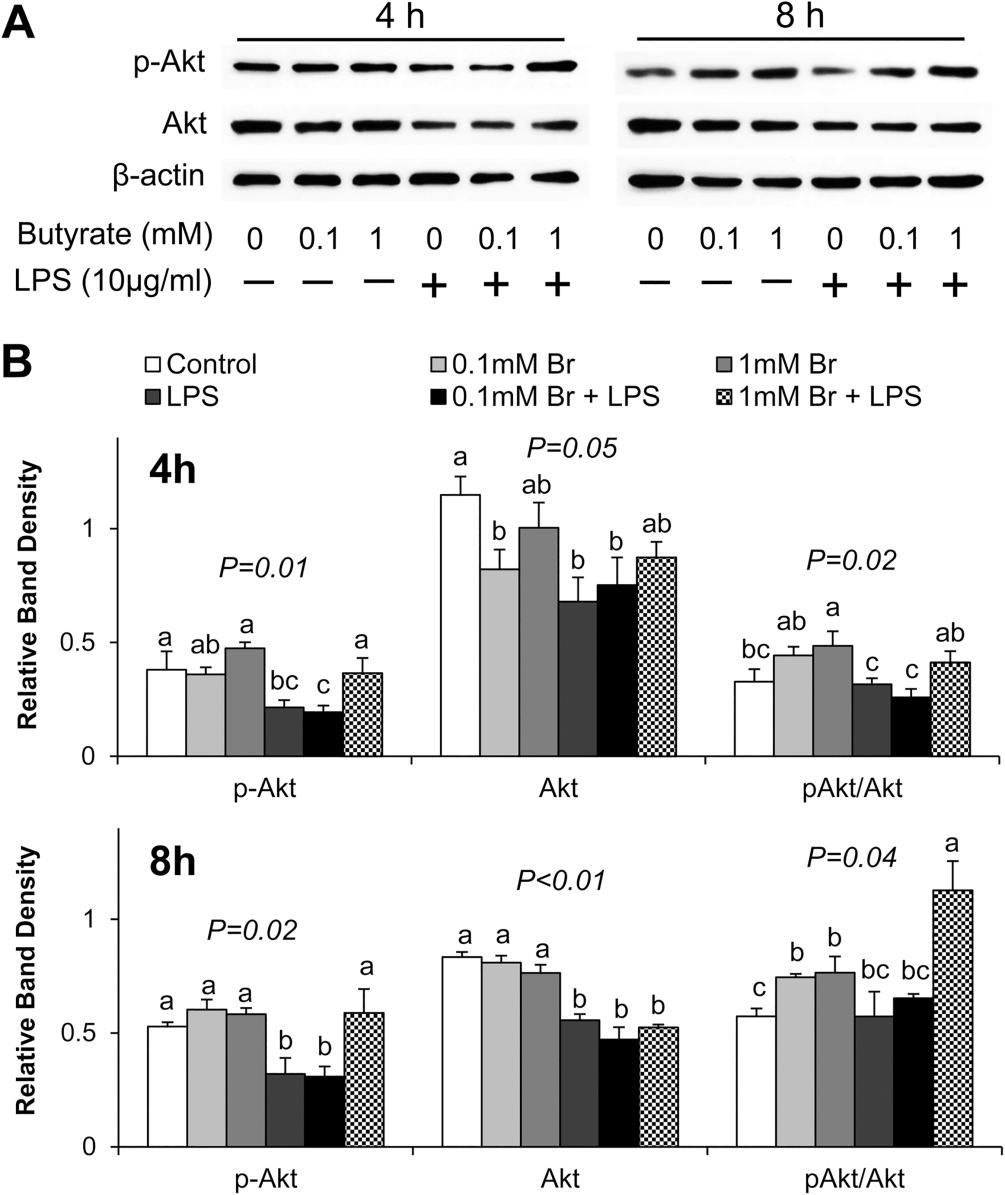
Effect of butyrate and LPS treatment on activation of Akt in IPEC-J2 cells. (A) Representative immunoblotting analysis of Akt after 4 and 8 h of LPS stimulation. (B) Quantification analysis of Akt abundance in IPEC-J2 cells. Data were analyzed by one-way ANOVA with Tukey multiple comparison test. Values are means ± SE, n = 4. Different superscript letters (a, b, c) on bars indicate significant mean differences, P < 0.05.

Because butyrate prevented LPS-induced downregulation of tight junction claudins, next we investigated potential effect of butyrate on the Akt/mTOR signaling pathway due to the significance of this pathway in regulating protein synthesis. LPS exposure resulted in significant reduction in the amount of phospho-Akt and total Akt proteins after 4 h and 8 h stimulation (P<0.05) (Fig 5). In contrast, butyrate treatment led to increases in pAkt/Akt ratio with or without LPS stimulation, and the higher concentration of butyrate (1mM) increased pAkt and pAkt/Akt ratio in LPS-treated cells (Fig 5). We noted that, although LPS reduced pAkt abundance and the pAkt/Akt ratio, (Fig 5), the higher concentration of butyrate (1mM) was able to prevent these decreases, indicating that butyrate, at the right concentration, might alleviate LPS-induced suppression of Akt signaling pathway.

**Fig 6:**
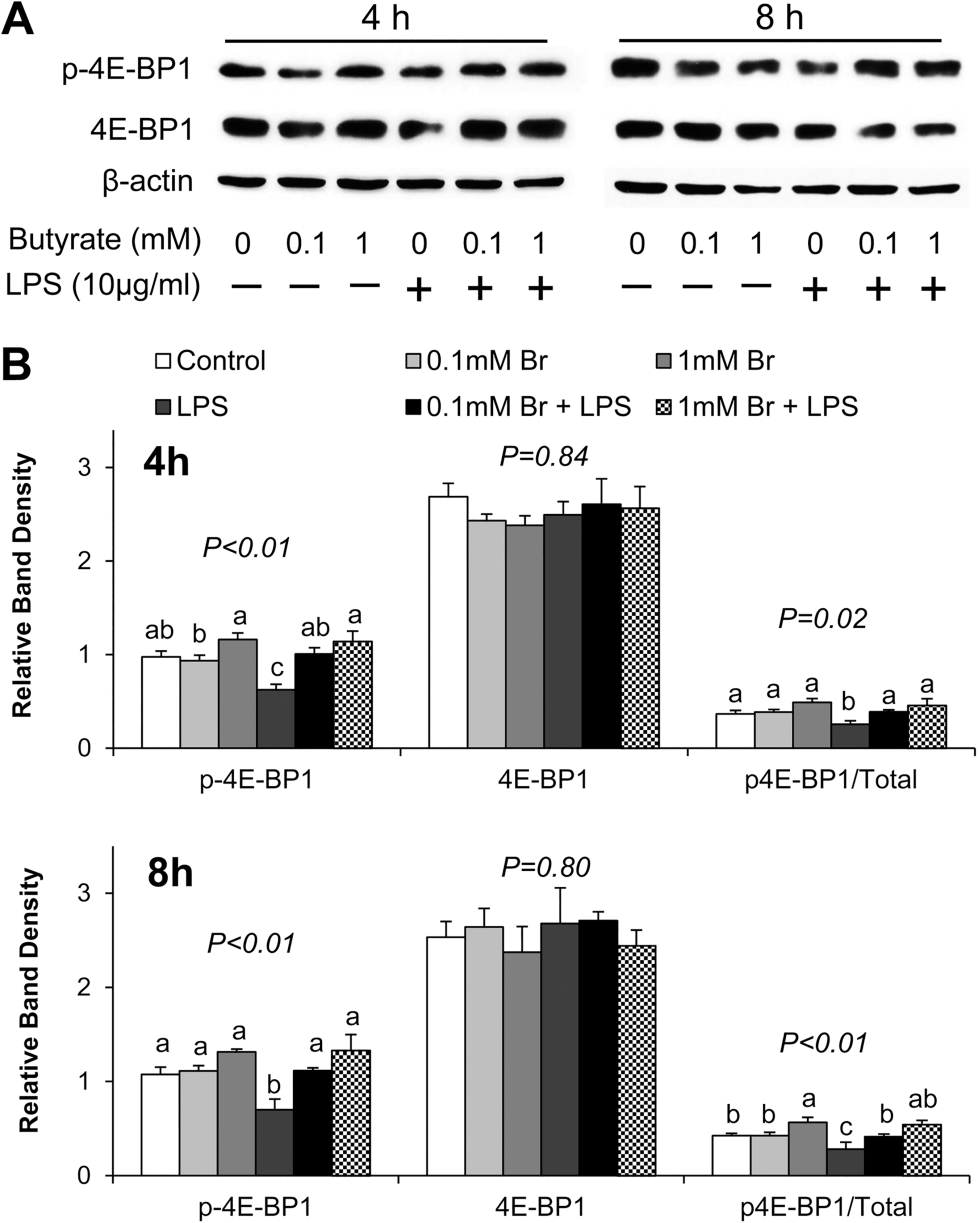
Effect of butyrate and LPS challenge on phosphorylation of 4E-BP-1 in IPEC-J2 cells. (A) Representative immunoblotting analysis of 4E-BP-1 after 4 and 8 h of LPS stimulation. (B) Quantification analysis of Akt abundance in IPEC-J2 cells. Data were analyzed by one-way ANOVA with Tukey multiple comparison test. Values are means ± SE, n = 4. Different superscript letters on bars (a, b, c) indicate significant mean differences, P < 0.05.

We further examined the phosphorylation of 4E-BP1, a downstream substrate of Akt/mTOR signaling. Phosphorylation of 4E-BP1 is known to lead to release of eukaryotic translation initiation factor 4E (eIF4E), which directly regulates mRNA translation. LPS-treated cells exhibited significantly lower phospho-4E-BP1 after 4 h and 8 h stimulation compared to control (Fig 6). In contrast, pretreatment of cells with both 0.1mM and 1mM butyrate increased the phospho-4E-BP1 abundance compared to the LPS only group, and thereby prevented LPS-induced decreases in the phosphorylation of 4E-BP1 (Fig 6).

Collectively, these findings demonstrate that LPS exposure leads to the reduction in activation of downstream Akt signaling substrate 4E-BP1, whereas butyrate reversed this reduction, potentially restoring LPS-induced inhibition of protein synthesis.

### Butyrate regulates intracellular ATP level and AMPK activation

**Fig 7:**
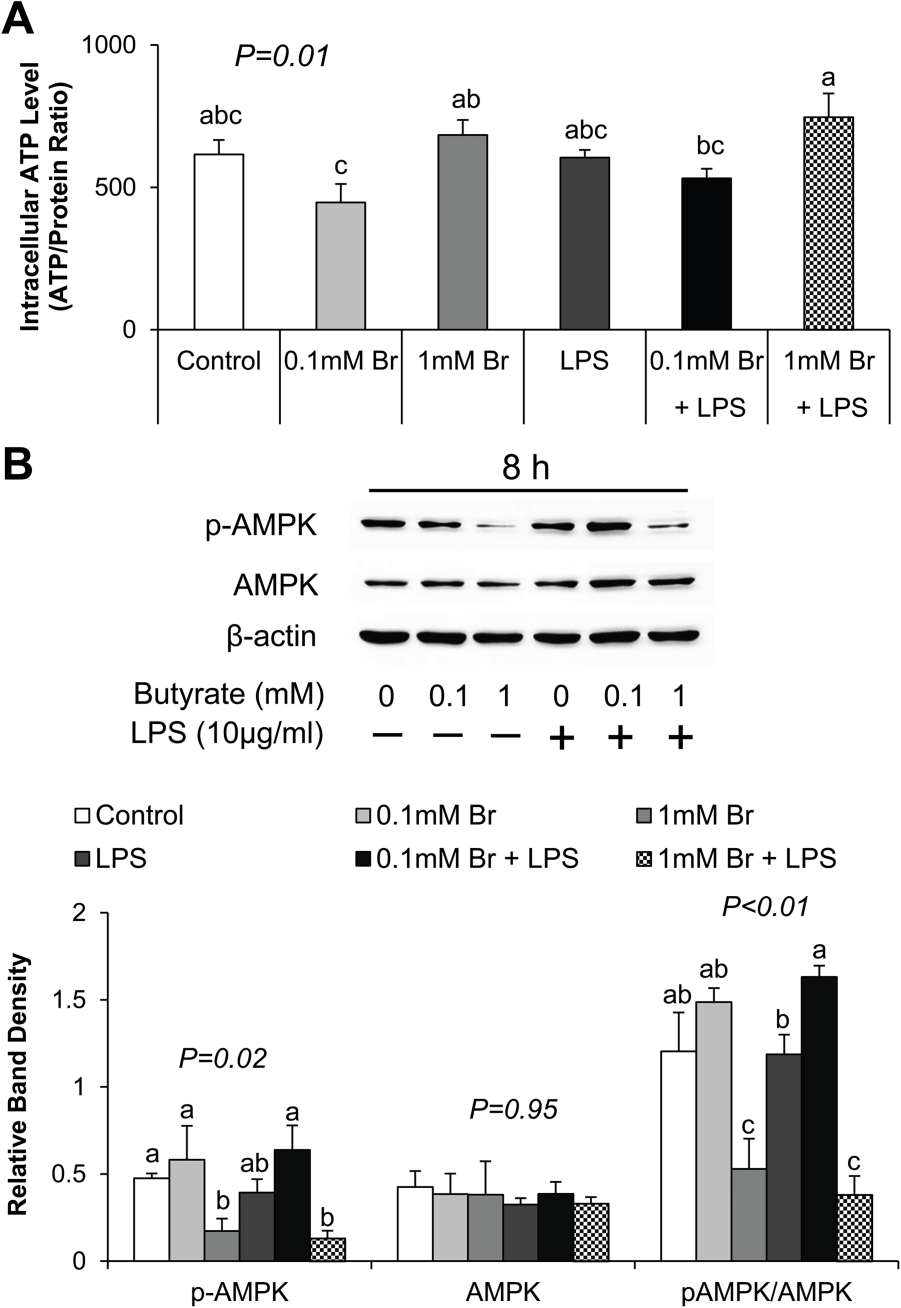
Effect of butyrate and LPS on intracellular ATP level and AMPK activation in IPEC-J2 cells. Cell lysates were isolated after 8 h of LPS challenge, and subjected to ATP determination assay and immunoblotting analysis. (A) Intracellular ATP level was measured by ATP determination kit and normalized to total protein concentration. (B) Representative immunoblotting and quantification analyses of AMPK abundance in IPEC-J2 cells. Data were analyzed by one-way ANOVA with Tukey multiple comparison test. Values are means ± SE, n = 4. Different superscript letters on bars (a, b, c) indicate significant mean differences, P < 0.05.

To determine whether butyrate serves as an energy source for the cells, we next measured intracellular ATP level after butyrate treatment and LPS challenge. Butyrate treatment influenced cellular ATP level in a dose-dependent manner, but LPS effect was not significant (Fig 7A). The concentration of ATP was significantly higher in cells treated with 1mM butyrate compared to those treated with 0.1 mM butyrate LPS (P<0.05) (Fig 7A), suggesting that butyrate dose may be important for its regulation of ATP concentration.

Intracellular ATP level is linked with AMPK activity. Therefore, we further examined AMPK protein abundance in response to LPS stimulation and butyrate treatment. Phosphorylation of AMPK is associated with a reduction in cellular ATP level. Butyrate at the higher concentration caused a reduction in phospho AMPK (indicated by the pAMPK/AMPK ratio), which agrees with the increase in cellular ATP concentration at this level of butyrate. Because these effects were not seen at the lower concentration of butyrate, these findings indicate that a sufficiently high concertation of butyrate could lead to an increase in the concentration of intracellular ATP level, leading to a downregulation of the level of activated AMPK.

## Discussion

The intestinal epithelial barrier selectively regulates epithelial permeability to luminal substances and antigens. The disruption of the intestinal barrier induced by toxins and pathogens contributes to the development of severe intestinal inflammation and digestive disorders [43, 44]. Short chain fatty acids, end products of bacteria fermentation in the large intestine, have been found to exert profound influences on intestinal barrier function, immune response, epithelial cell proliferation and bacterial pathogenesis [15, 21]. In the present study, we demonstrate that butyrate enhances intestinal barrier integrity against LPS-induced impairment. The IPECJ-2 cell model is a well-established model for studying intestinal barrier function [26, 45]. In preliminary studies, we confirmed the optimal butyrate concentration for these cells to range from 0.1 mM to 1 mM as these concentrations did not cause a significant impairment of cell viability. Others have reported that butyrate promoted intestinal barrier integrity at about 2 mM in Caco-2 cells [25], a much higher concentration than used in the present study. Caco-2 cells are a transformed human colonic cell line whereas IPEC-J cells are untransformed porcine jejunal cells. Thus, differences in cellular origin might be responsible for the lower tolerance of IPEC-J cells to butyrate compared to Caco-2. Although colonic cells are very tolerant of high butyrate concentration, exposure of these cells to concentrations higher than 3 mM induced cytotoxicity and apoptosis through activation of mitochondrial damage [46] and JNK/AP1 pathway [47].

In the present study, we demonstrate that butyrate promotes intestinal barrier integrity, indicated by the increases in TEER and decreases in paracellular permeability in butyrate treated cells. Although IPEC-J2 cells are vulnerable and sensitive to LPS-induced inflammation and impairment of intestinal epithelial integrity [27, 28], pretreatment of cells with 1 mM butyrate, significantly mitigated LPS effect. Intestinal barrier integrity is maintained by the tight junctions, made of transmembrane, scaffold and adaptor proteins. Claudins and occludin are transmembrane proteins embedded in the intracellular actin through attachment to adaptor protein ZO-1 [16]. In the present study, we found that butyrate increased the expression of claudin-3, claudin-4 and restored the reduction in the abundance of these proteins caused by LPS challenge. Claudins are the main structural and functional components of tight junctions, selectively preventing passage of luminal substances through paracellular routes [16]. Decrease in protein expression of claudins is highly correlated to impaired intestinal barrier function [41, 48]. Therefore, the protective effect of butyrate on intestinal barrier against LPS damage may be partly explained by the increase in the expression of claudin proteins. However, the observed effect on tight junction proteins is selective because neither LPS nor butyrate had any significant influence on the expression of occludin. Occludin is known to regulate the gating of tight junctions [48–50]. However, disruption of occludin expression and function does not lead to failure of the intestinal barrier [48], confirming that occludin may be dispensable for the maintenance of intestinal barrier integrity. ZO-1 couples claudins and occludin to cytoplasmic proteins and to the actin cytoskeleton [48]. In Caco-2 cells, severe inflammation leads to a reduction in ZO-1 expression and the impairment of barrier function, as measured by the TEER and the flux of dextran [51]. Although a reduction of ZO-1 expression by LPS was confirmed in the present study, unlike in the case of claudin-3, claudin-4, no effect of butyrate was observed. This suggests a selective regulation of tight junction proteins by butyrate. The mechanism of this selective regulation of tight junction proteins by butyrate is presently unknown.

LPS activates inflammatory pathway through binding and activation of TLR-4, its cognate receptor [30, 31]. We show here that butyrate downregulates the expression of TLR-4. Thus, downregulation of expression of TLR-4 may partly explain the suppression of LPS effect by butyrate. The inflammatory cytokines are secondary mediators of LPS-induced damage of tight junctions and impairment of epithelial cell integrity [32, 33, 52]. Indeed, multiple inflammatory cytokines have been shown to disrupt epithelial tight junction and barrier integrity [48]. The interleukins, TNFα and interferon gamma (IFNγ), are known to increase epithelial cell permeability through impairment of tight junction assembly and actin cytoskeletal structure [32, 33, 53]. Paradoxically, we also found that the higher concentration of butyrate can also evoke an inflammatory response because it induced IL-8 mRNA expression and protein secretion. However, butyrate has also been shown to induce IL-8 secretion in Caco-2 cells [54]. The observed mild selective cytokine induction by butyrate may be part of its mechanism of intestinal protection, as this may contribute to an enhancement of epithelial immune defense, rather than an impairment of epithelial integrity. However, the mechanism of butyrate upregulation of IL-8 remains to be determined.

Enhancement of tight junction protein expression is highly associated with the activation of Akt signaling pathway in intestinal epithelial cells [55]. The Akt/mTOR mediated protein synthesis pathway is implicated in the promotion of cellular growth [56]. Activation of Akt phosphorylates mTOR and leads to activation of downstream substrates, p70S6K1 and 4E-BP1, which directly regulates protein synthesis [57]. In addition, Akt pathway plays an important role as a negative feedback regulator of LPS-induced inflammatory response [34–36]. The Akt pathway is involved in the regulation of various cellular functions, including cell survival, growth, proliferation and metabolism [57]. In the present study, LPS stimulation resulted in decreases in phospho-Akt and total Akt abundance, and subsequently led to a reduction of phospho-4E-BP1 abundance, indicating LPS inhibits Akt/mTOR signaling pathway and perhaps protein synthesis, and this potentially accounts for the LPS-induced disruption of epithelial barrier. However, treatment of IPEC-J2 cells with butyrate negated the LPS-induced decreases in phospho-Akt and phospho-4E-BP1 protein abundance, indicating that butyrate might exert its protective effect through up-regulation of Akt signaling pathway to counter the LPS-induced reduction in tight junction protein synthesis.

Butyrate has been shown to enhance the intestinal barrier function through activation of AMPK in Caco-2 cells [24]. However, the underlying mechanism is still unclear. The observed activation of AMPK with the low dose of butyrate (0.1 mM) will be in agreement with the Caco-2 study [24]. On the contrary, the higher level of butyrate (1 mM) led to a reduction in AMPK phosphorylation. Thus, effect of butyrate on AMPK phosphorylation and activation status is concentration dependent. Explanation for this observation appears related to the effect of the different concentrations of butyrate on cellular ATP level. AMPK is an energy sensor that regulates metabolic pathways, including metabolism of glucose and fatty acid and protein synthesis [58]. However, AMPK phosphorylation status and activity are modulated by the changes in ATP: AMP ratio. A high level of intracellular ATP and an increase in the ATP:AMP ratio leads to an inactivation of AMPK, and *vice versa* [59]. In this study, we found that intracellular ATP level was decreased by the low concentration butyrate whereas it was increased by the high concentration of butyrate (1 mM), and these results agree with increase in AMPK phosphorylation at the low butyrate level and the decrease at the high concentration. We surmise that, although both butyrate levels enhance intestinal epithelial barrier and tight junction expression through activation of Akt and 4E-BP1, the higher butyrate level was able to supply sufficient substrate for energy generation to drive this energetically expensive process, subsequently leading to higher cellular ATP (hence lower AMPK phosphorylation). However, the lower level of butyrate could not provide enough energy to support the stimulation of tight junction protein synthesis, leading to depletion of cellular ATP, hence increased AMPK phosphorylation.

In conclusion, we have demonstrated that butyrate restored LPS-induced impairment of the intestinal barrier by promoting tight junction (especially claudins) protein expression, perhaps via activation of Akt-mediated protein synthesis pathway in IPEC-J2 cells. Results obtained agree with the observed enhancement of epithelial barrier integrity in CACO-2 cells by butyrate [60]. Although we could not rule out the potential involvement of other signaling pathways such as the Erk1/2 [61] in the mediation of butyrate effect, however, this possibility was not investigated in this study. The observed butyrate regulation of AMPK phosphorylation and activation may result from its role in the regulation of energy homeostasis, agreeing with the recognized importance of butyrate as an energy source for epithelial cell proliferation and maintenance of intestinal barrier integrity.

## Supporting information

S1 Fig Cell viability in IPEC-J2 cells treated with LPS or butyrate. IPEC cells were treated with or without LPS (10ug/ml) or indicated concentrations of butyrate for 24 hours. The Cell Quanti-Blue cell viability assay kit (BioAssay Systems, Hayward, CA) was then used to measure viability. No significant differences in viability was obtained across treatments.
(PDF)

S2 Fig Raw western blot images. Uncropped images of western blots are displayed as shown.
(DOCX)

S1 Text Raw primary data points used for calculations. Raw data used for data summaries are presented as shown.
(XLSX)

## Abbreviations

AMPK: AMP-activated protein kinase
Br: butyrate
FITC-dextran: fluorescein isothiocyanate-dextran
GIT: gastrointestinal tract
IL: interleukin
IPEC-J2: intestinal porcine epithelial cell line
LPS: lipopolysaccharide
MCP-1: monocyte chemoattractant protein
mTOR: mechanistic target of rapamycin
pAkt: phosphorylated Akt
pAMPK: phosphorylated AMP-activated protein kinase
p4E-BP1: phosphorylated eukaryotic translation initiation factor 4E-binding protein 1
TEER: trans-epithelial electrical resistance
TLR-4: toll like receptor -4
TNFα: tumor necrosis factor alpha
ZO-1: zonula occludens -1
4E-BP1: eukaryotic translation initiation factor 4E-binding protein 1.
