# Supplementary figures and images for: Butyrate modifies intestinal barrier function in IPEC-J2 cells through a selective upregulation of tight junction proteins and activation of the Akt signaling pathway
Source: PLoS One. 2017 Jun 27;12(6):e0179586. doi: 10.1371/journal.pone.0179586 (PMC5487041; doi:10.1371/journal.pone.0179586)

Relative Viability (%)

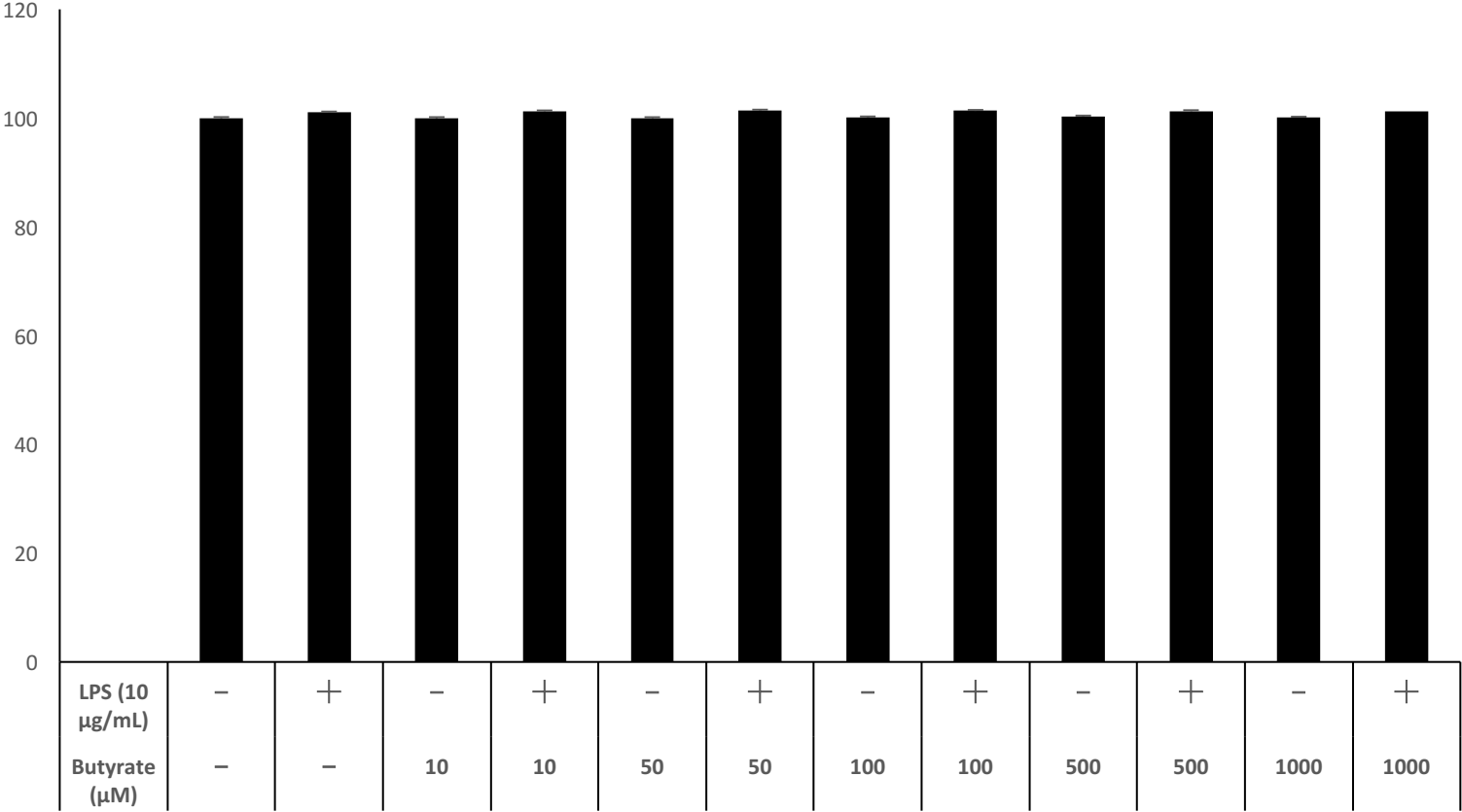

# Raw Data

|    | Butyrate ( $\mu\text{M}$ ) | LPS (10 $\mu\text{g/mL}$ ) | Viability | SE          |
|----|----------------------------|----------------------------|-----------|-------------|
| 1  | –                          | –                          | 100       | 0.18515106  |
| 2  | –                          | +                          | 101.009   | 0.184082898 |
| 3  | 10                         | –                          | 99.9948   | 0.189161484 |
| 4  | 10                         | +                          | 101.195   | 0.175655194 |
| 5  | 50                         | –                          | 100.025   | 0.20666094  |
| 6  | 50                         | +                          | 101.3498  | 0.13520682  |
| 7  | 100                        | –                          | 100.168   | 0.198648057 |
| 8  | 100                        | +                          | 101.321   | 0.126712367 |
| 9  | 500                        | –                          | 100.2982  | 0.172885159 |
| 10 | 500                        | +                          | 101.2543  | 0.157030742 |
| 11 | 1000                       | –                          | 100.117   | 0.192609258 |
| 12 | 1000                       | +                          | 101.293   | 0.130912862 |

Supplement: S1 Fig — IPEC cells were treated with or without LPS (10ug/ml) or indicated concentrations of butyrate for 24 hours. The Cell Quanti-Blue cell viability assay kit (BioAssay Systems, Hayward, CA) was then used to measure viability. No significant differences in viability was obtained across treatments. (PDF) [file pone.0179586.s001.pdf]
