# Supplementary material for: Butyrate modifies intestinal barrier function in IPEC-J2 cells through a selective upregulation of tight junction proteins and activation of the Akt signaling pathway
Source: PLoS One. 2017 Jun 27;12(6):e0179586. doi: 10.1371/journal.pone.0179586 (PMC5487041; doi:10.1371/journal.pone.0179586)

Extended Data Fig 3A

4h

20kDa

Claudin-3


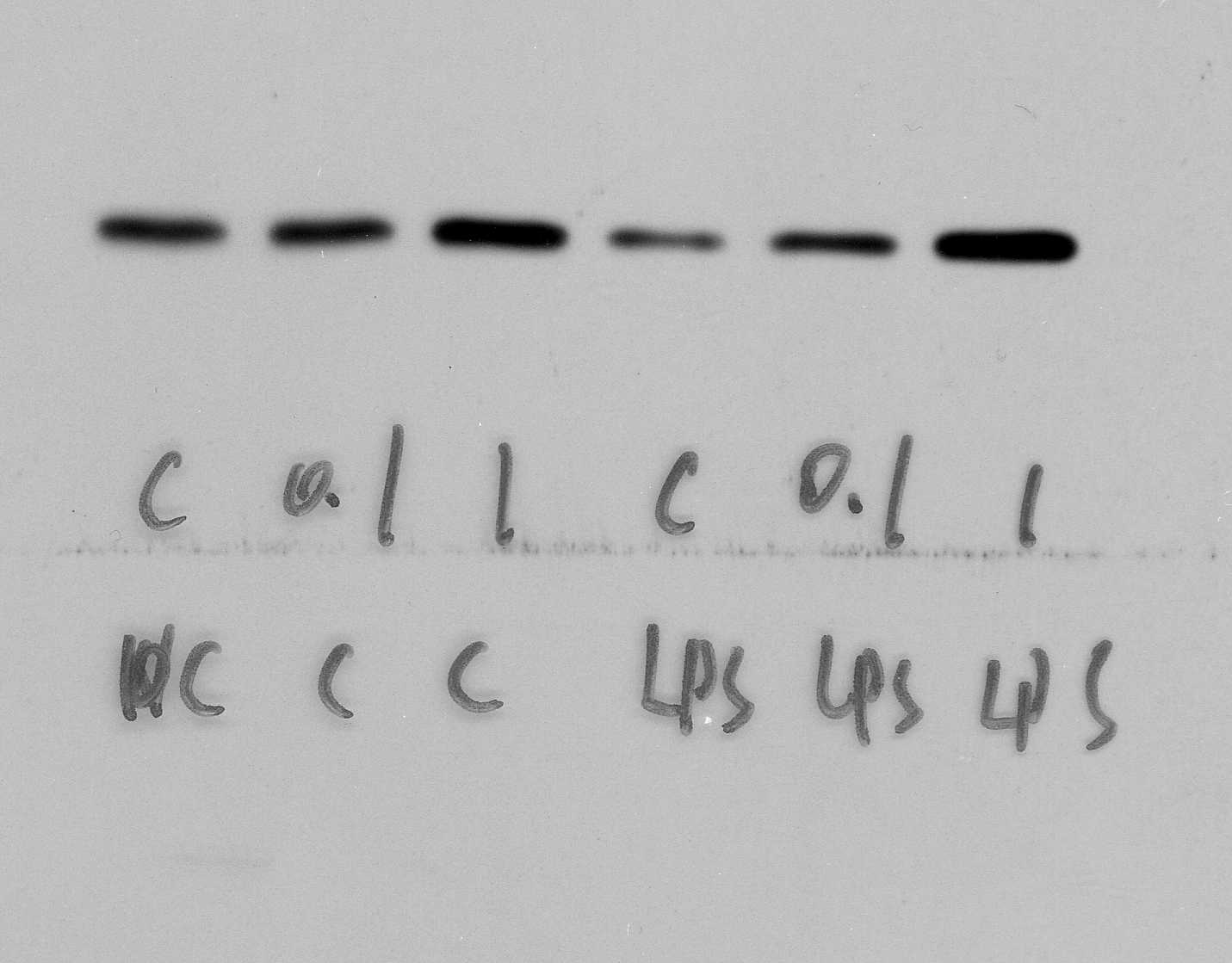


20kDa

Claudin-4


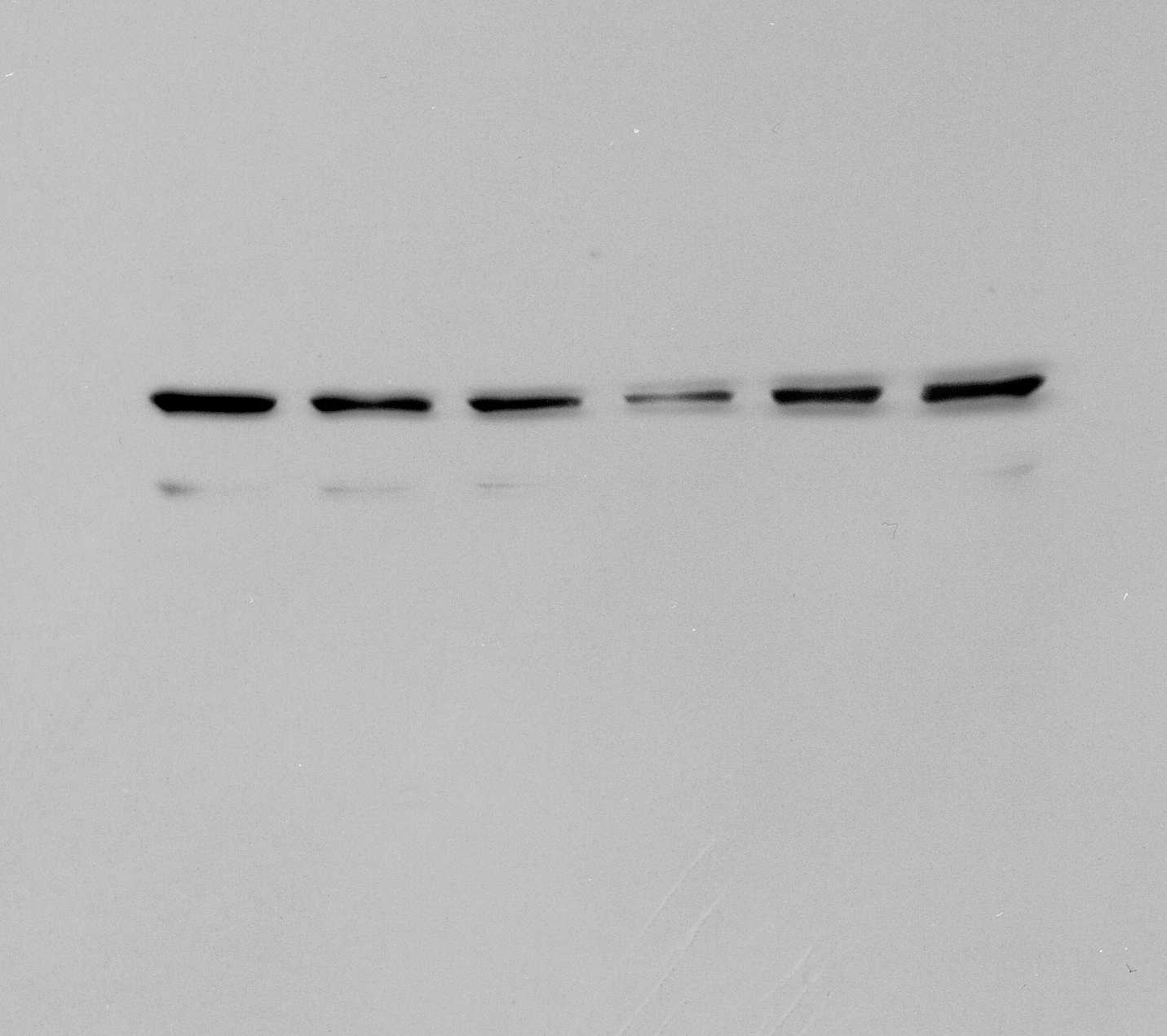


200kDa

ZO-1


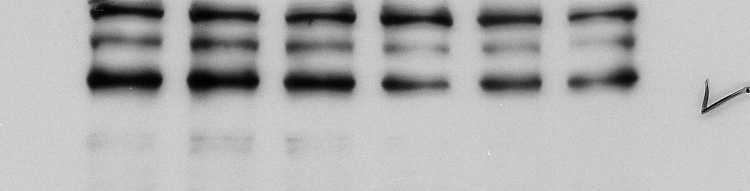


40kDa

β-actin


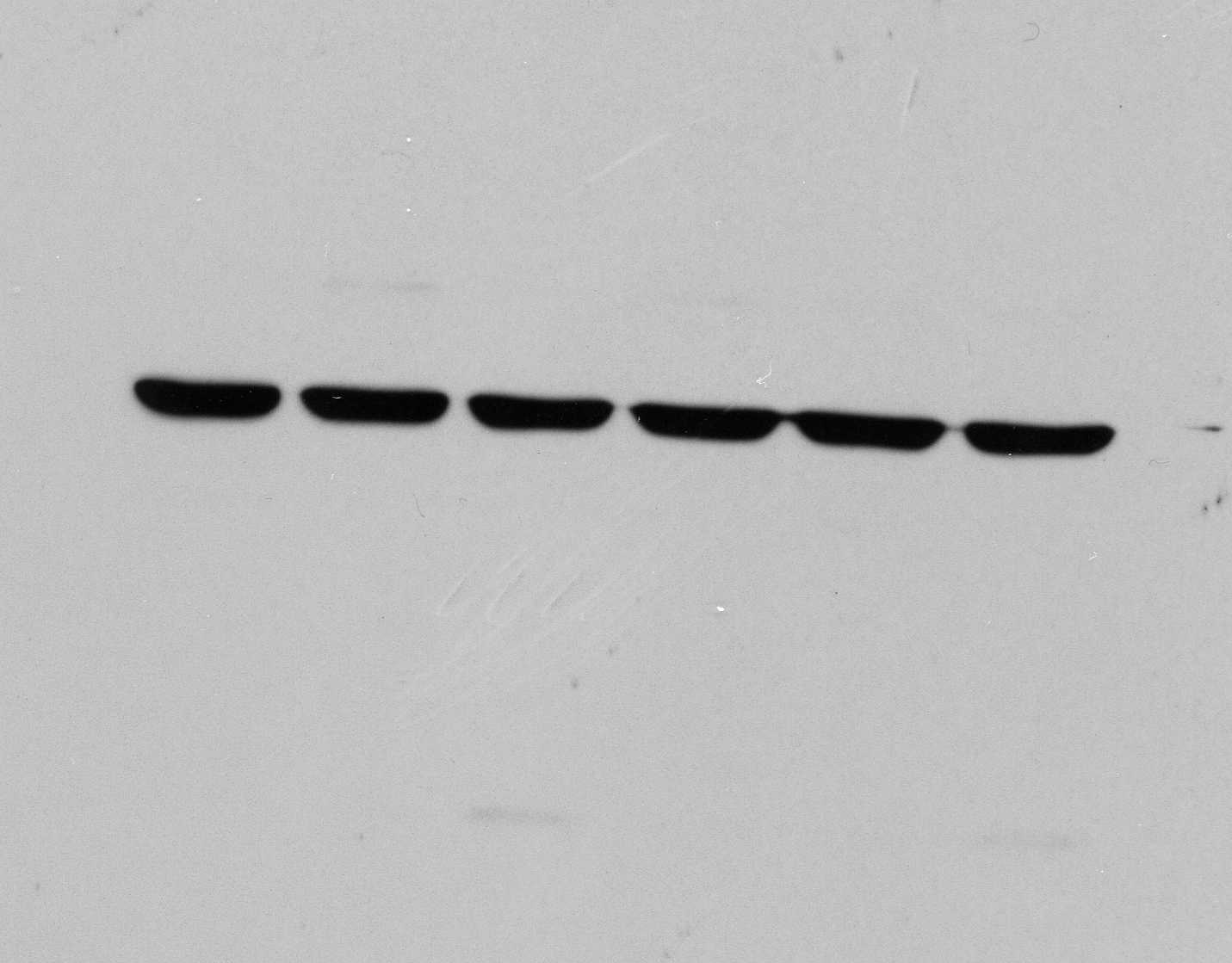


8h

20kDa

Claudin-3


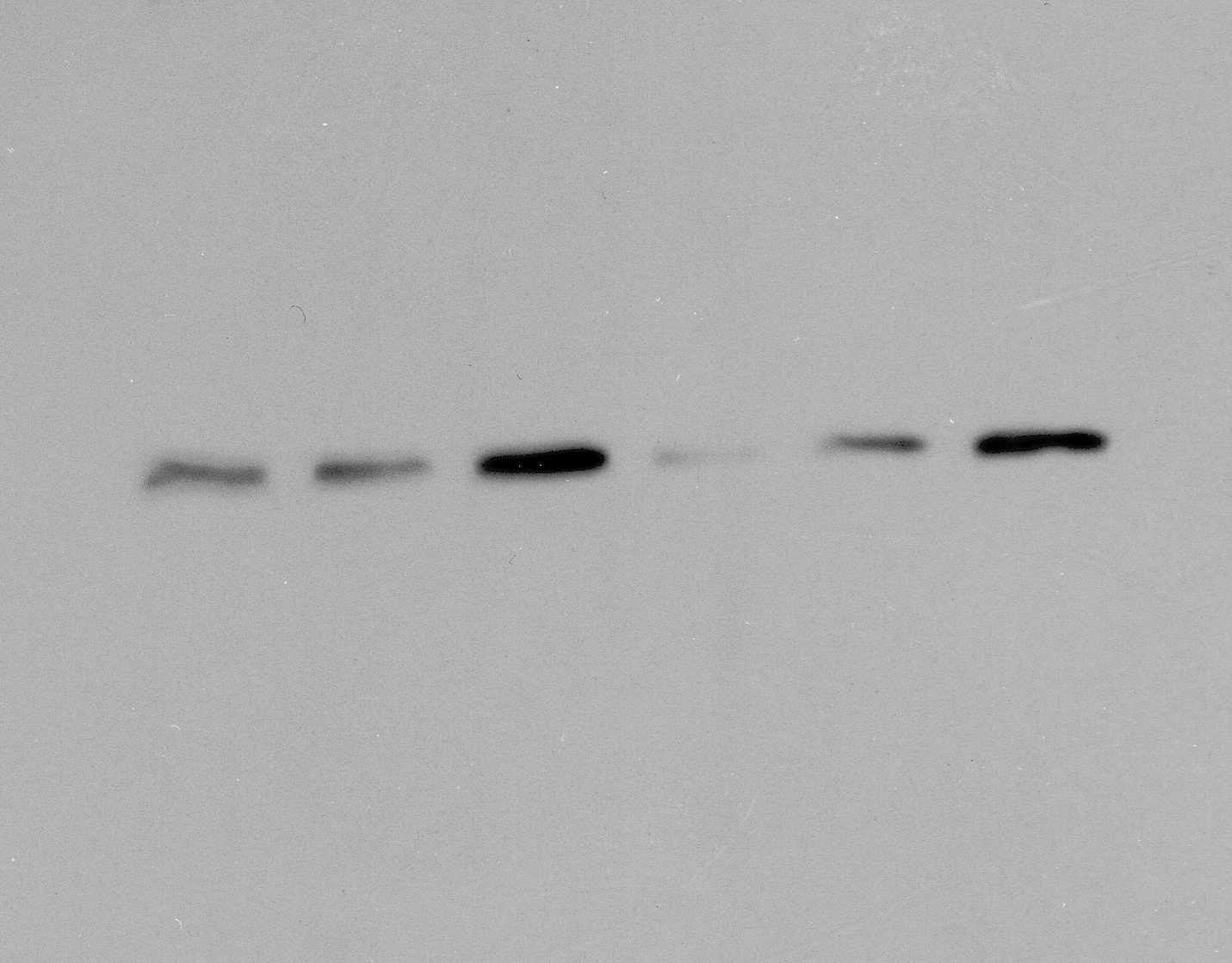


20kDa

Claudin-4


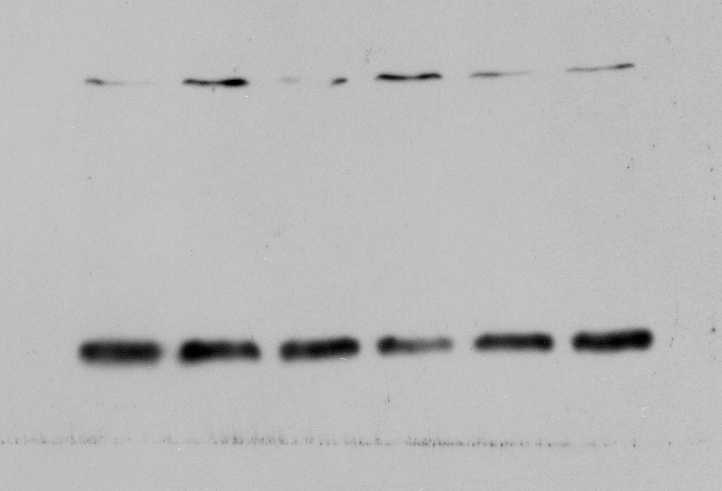


200kDa

ZO-1


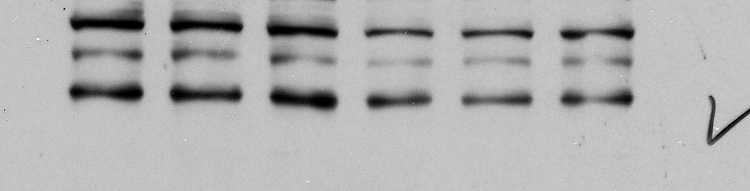


40kDa

β-actin


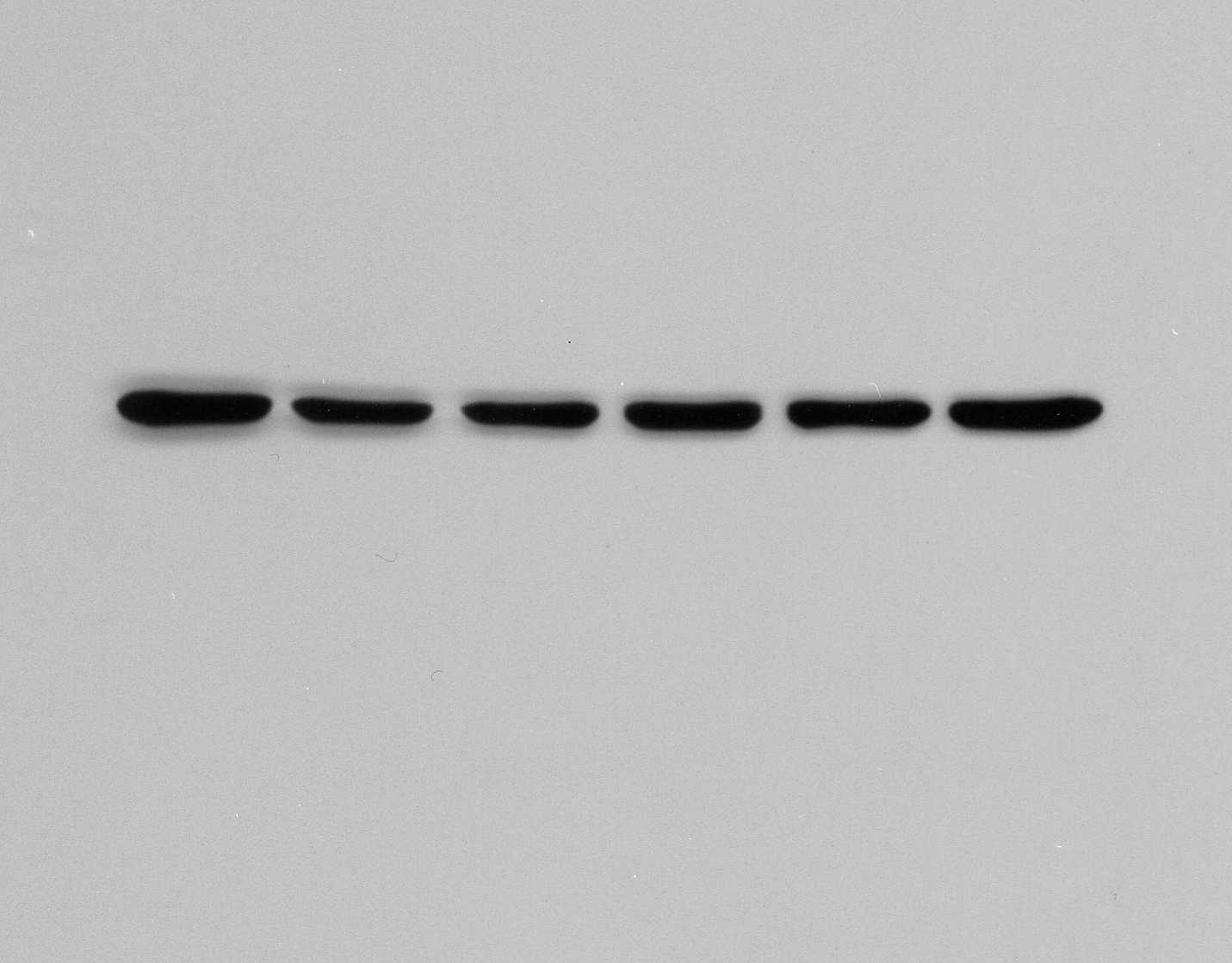


Extended Data Fig 3C

4h

20kDa

Claudin-1


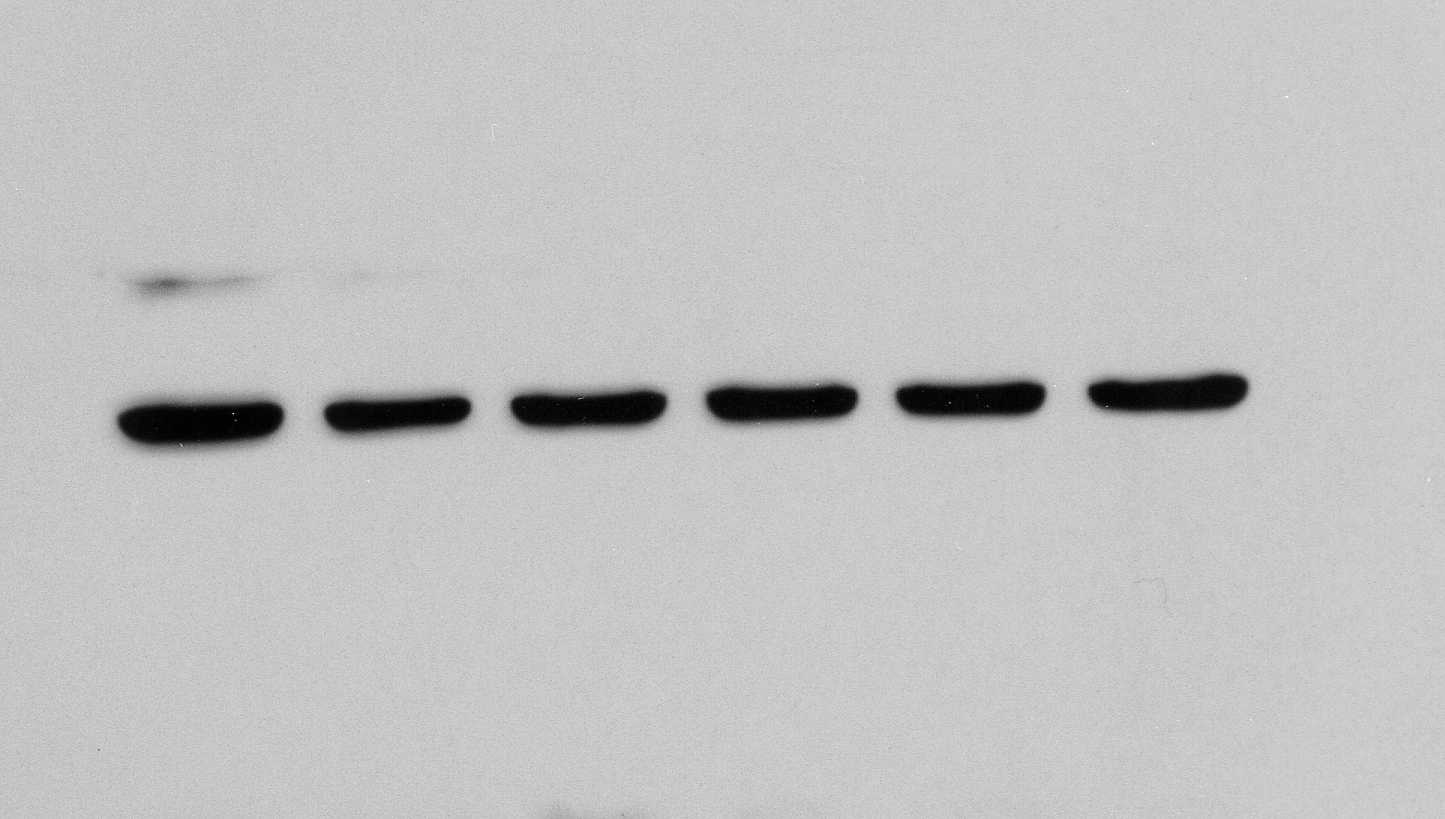


60kDa

50kDa

Occludin


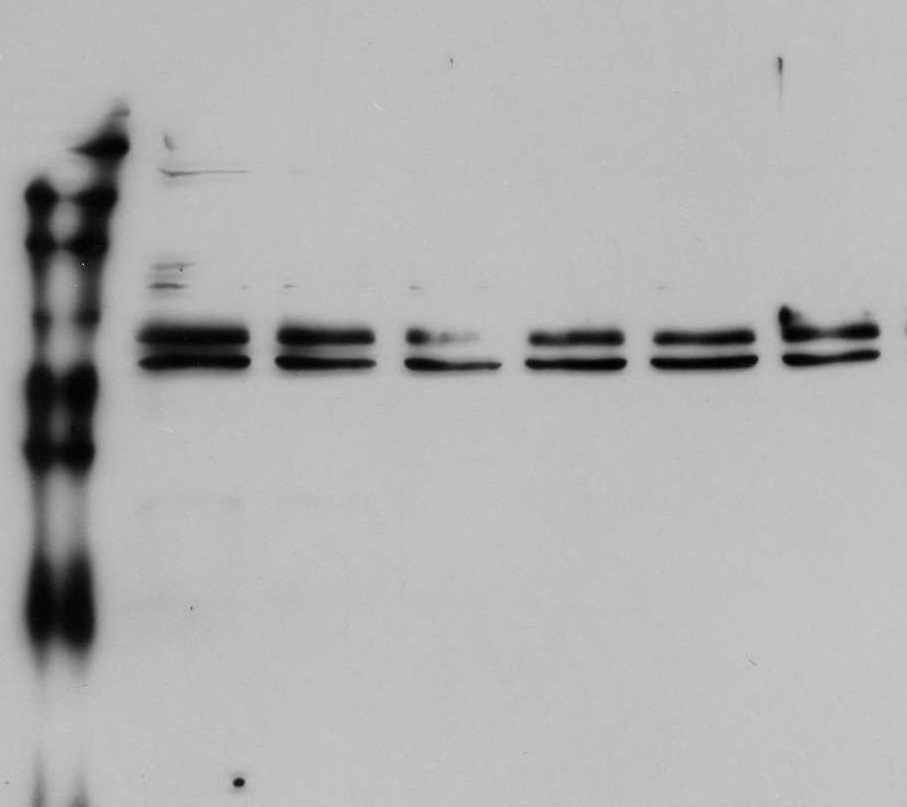


40kDa

β-actin


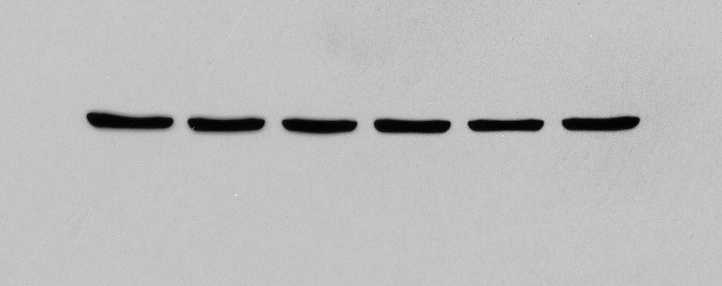


8h

20kDa

Claudin-1


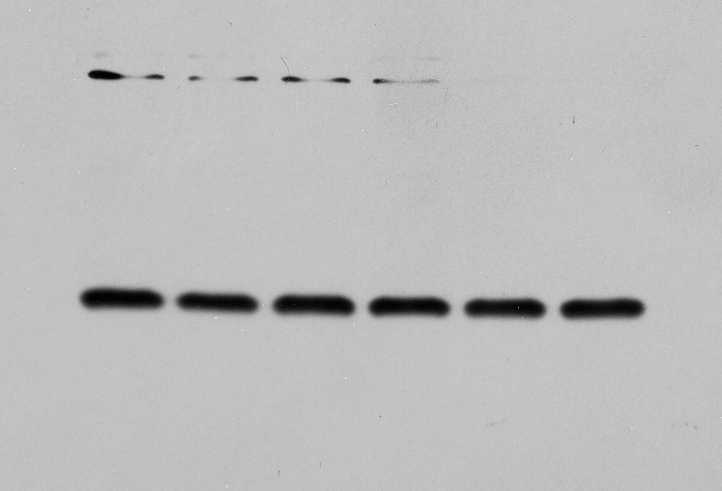


50kDa

Occludin


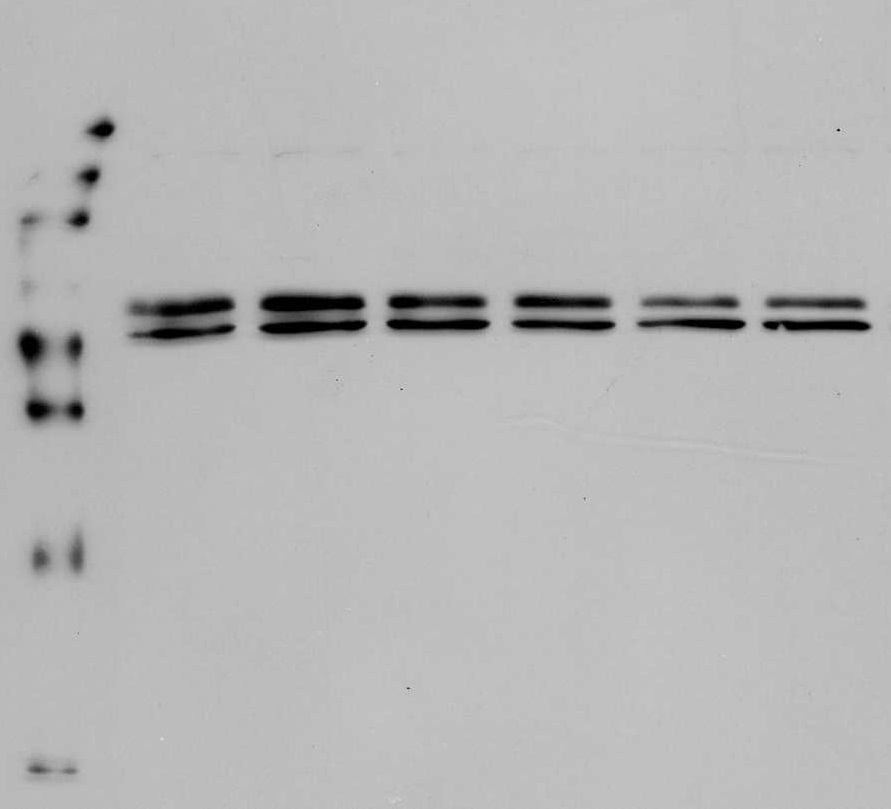


40kDa

β-actin


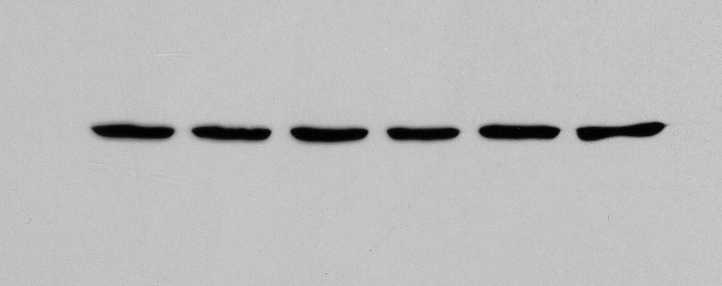


Extended Data Fig 5A

4h

60kDa

p-Akt


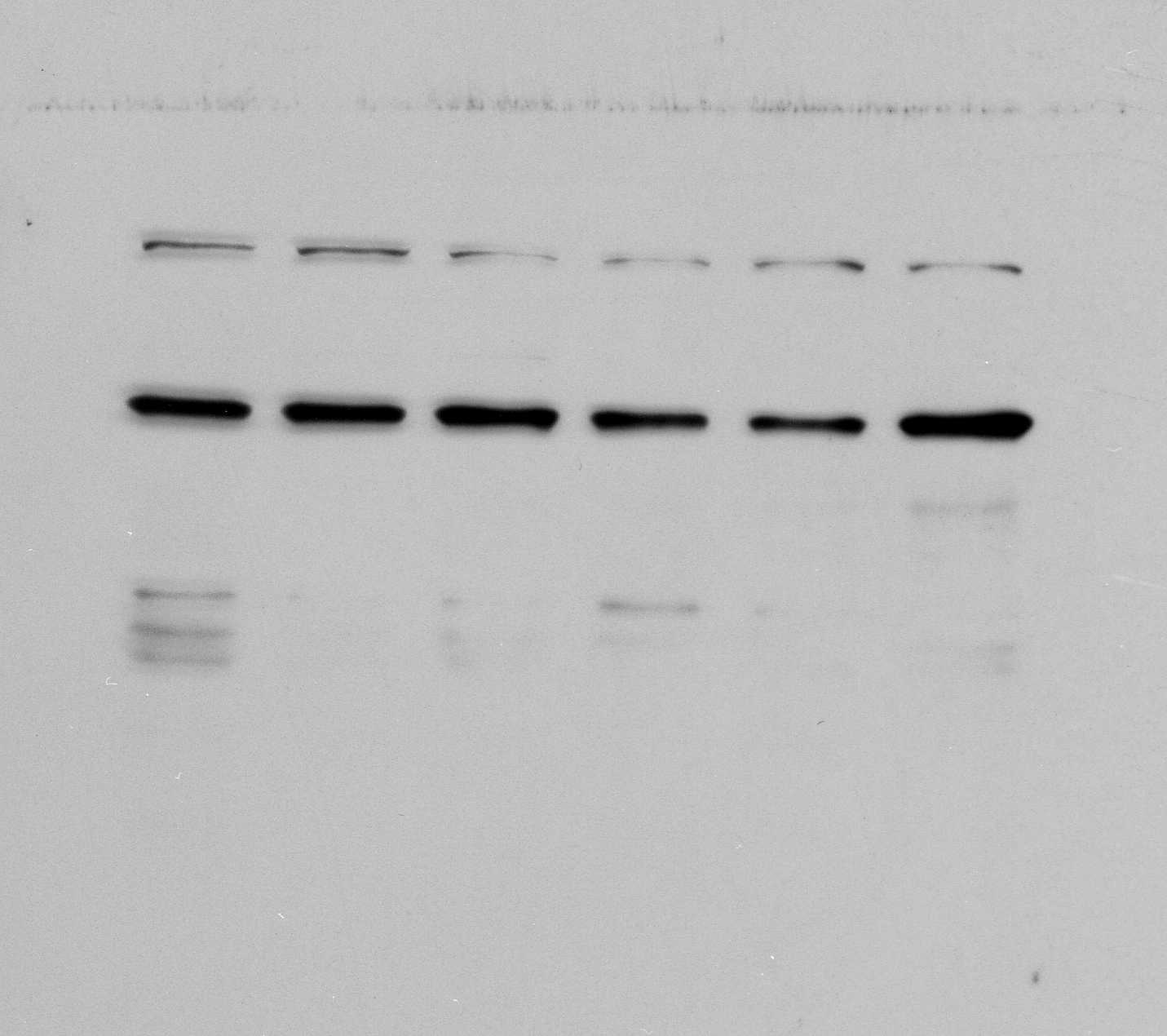


60kDa

Akt


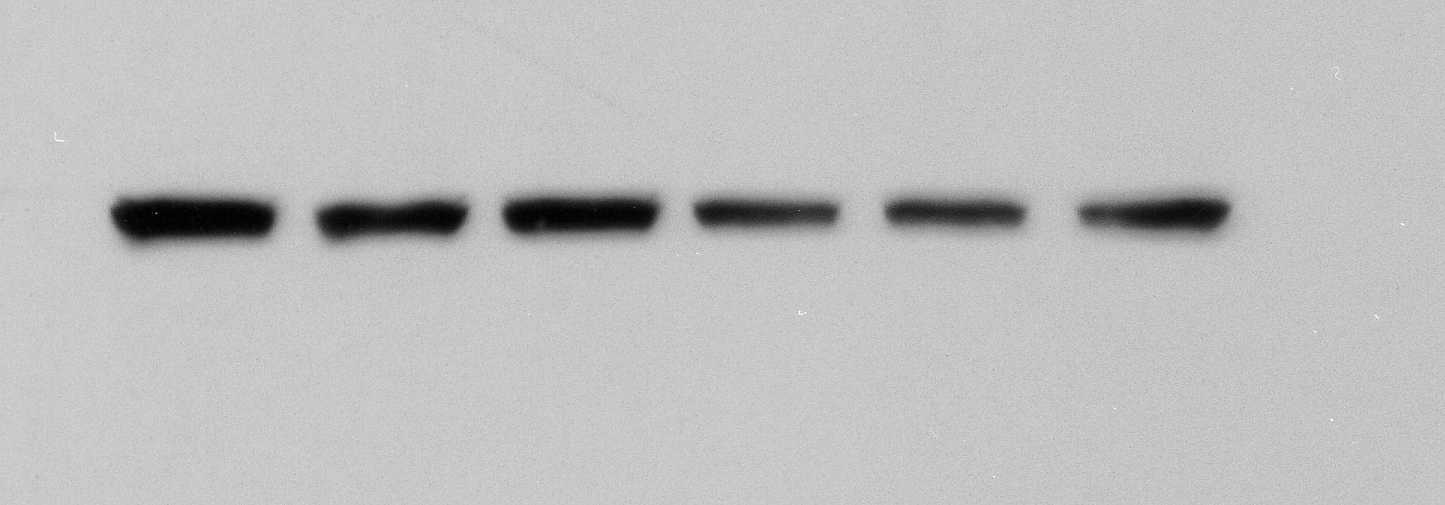


40kDa

β-actin


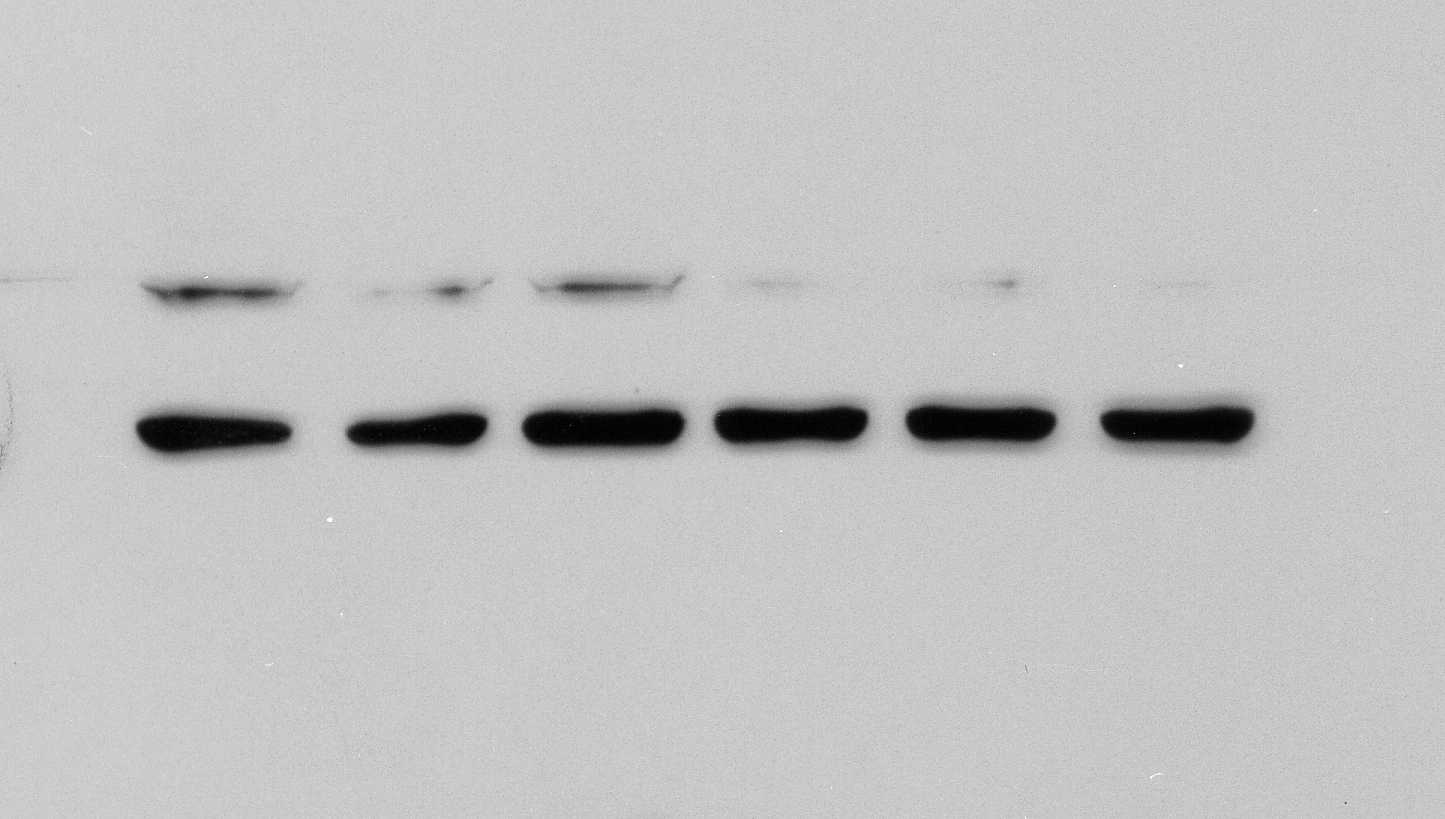


8h

60kDa

p-Akt


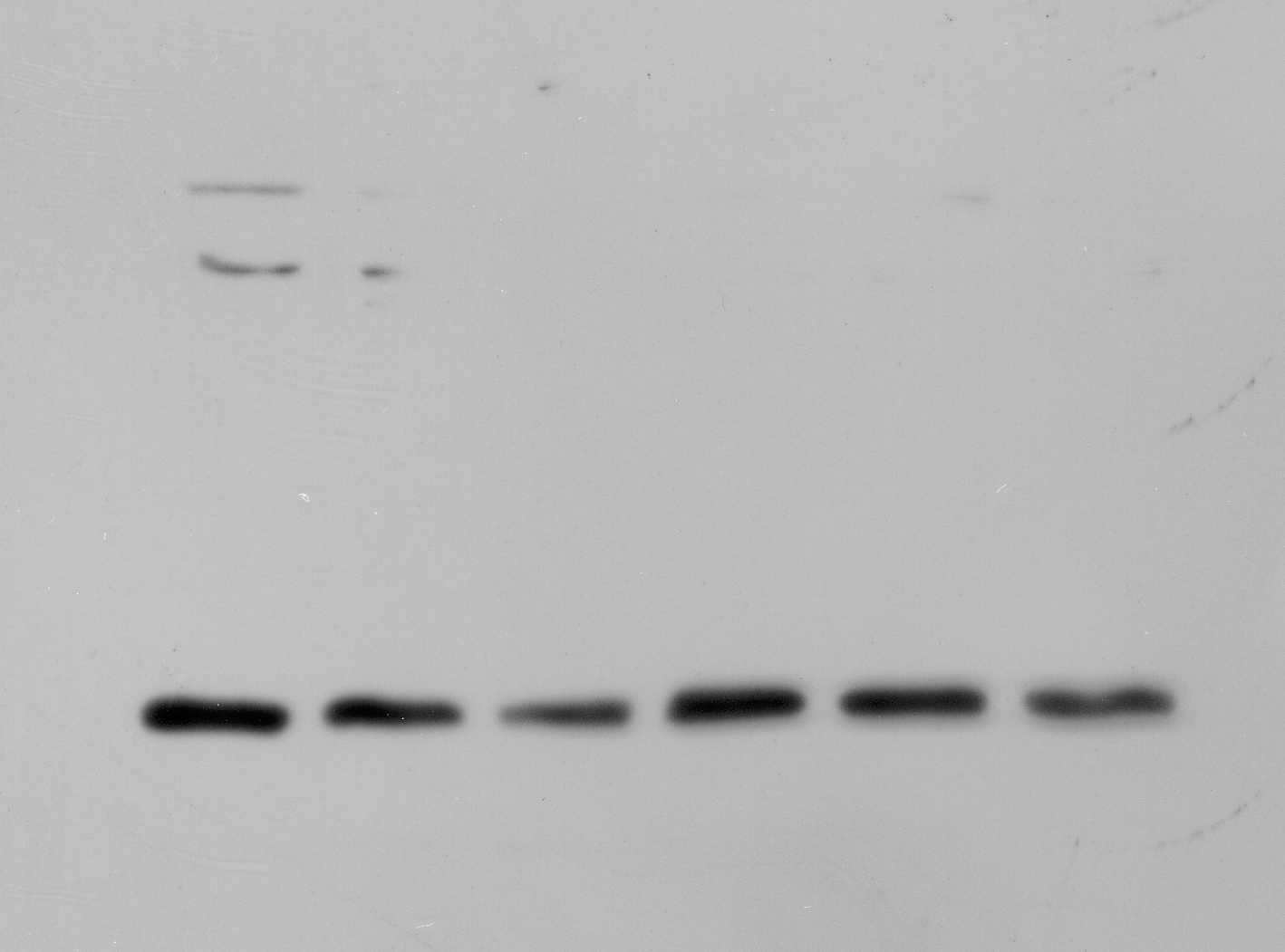


60kDa

Akt


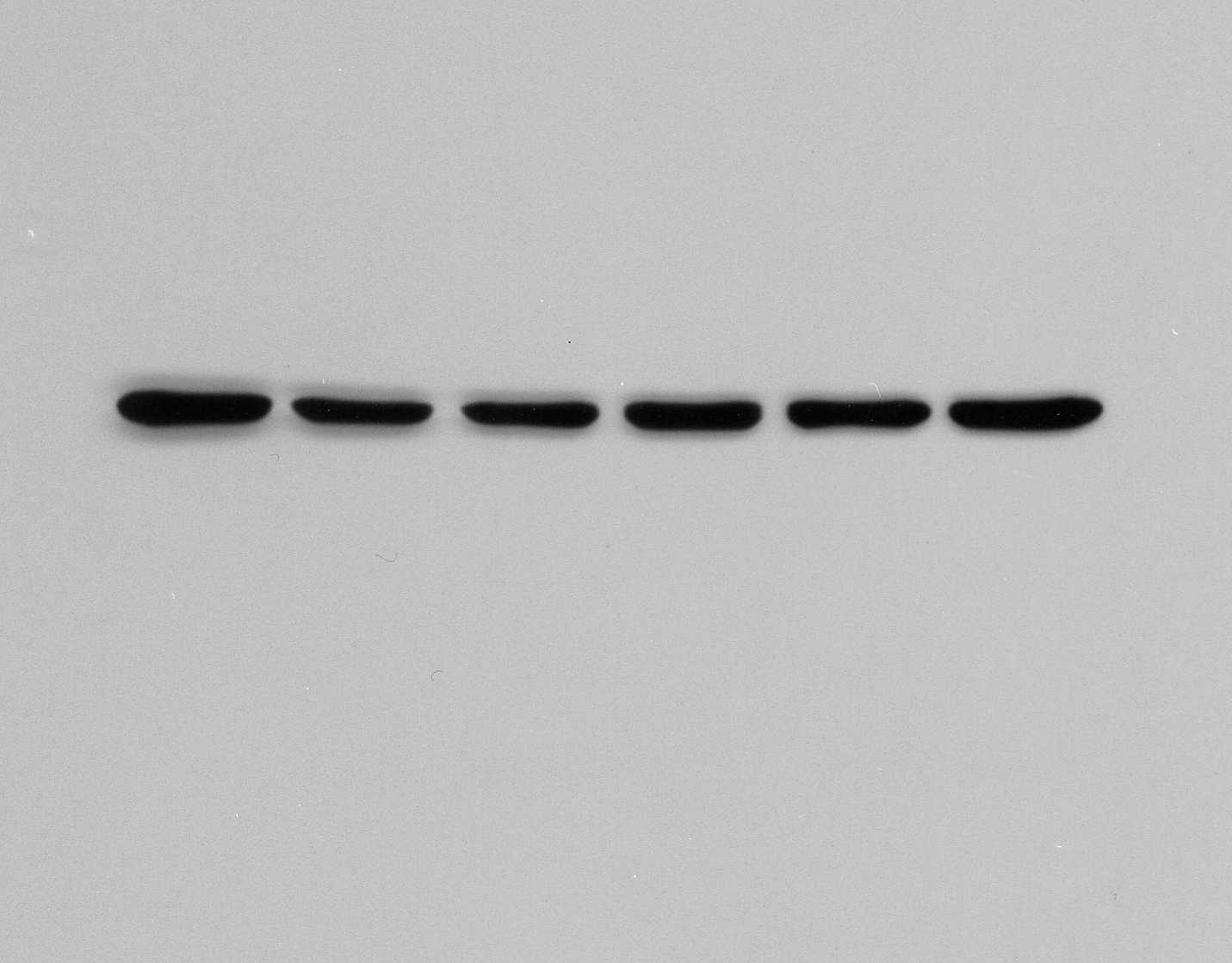


40kDa

β-actin


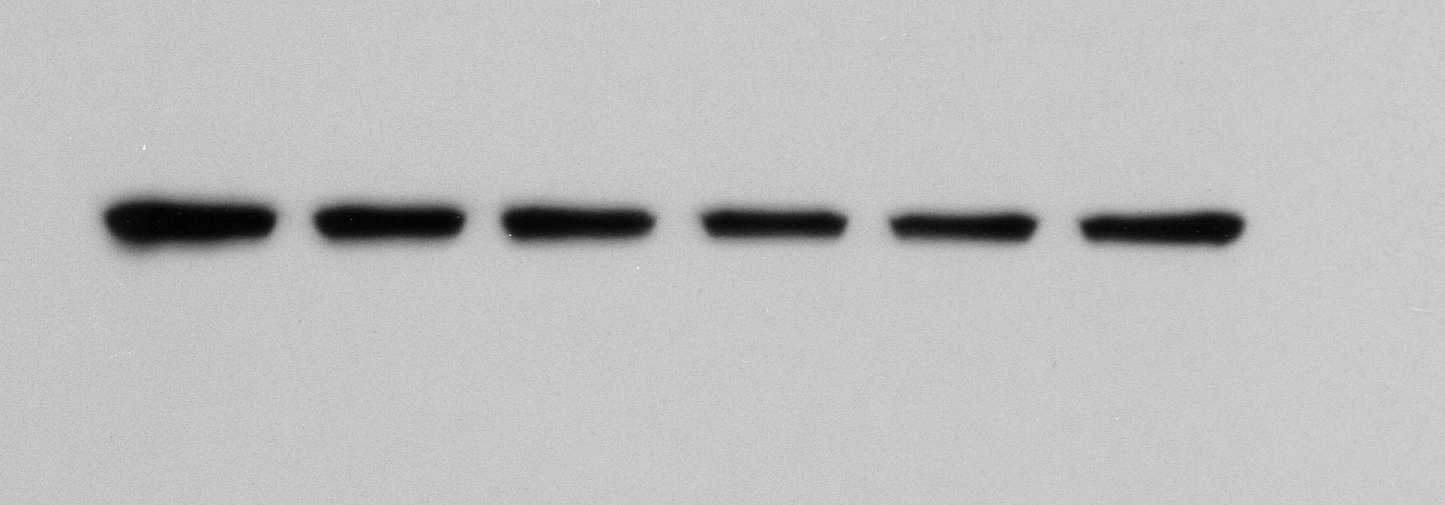


Extended Data Fig 6A

4h

20kDa

p-4E-BP1


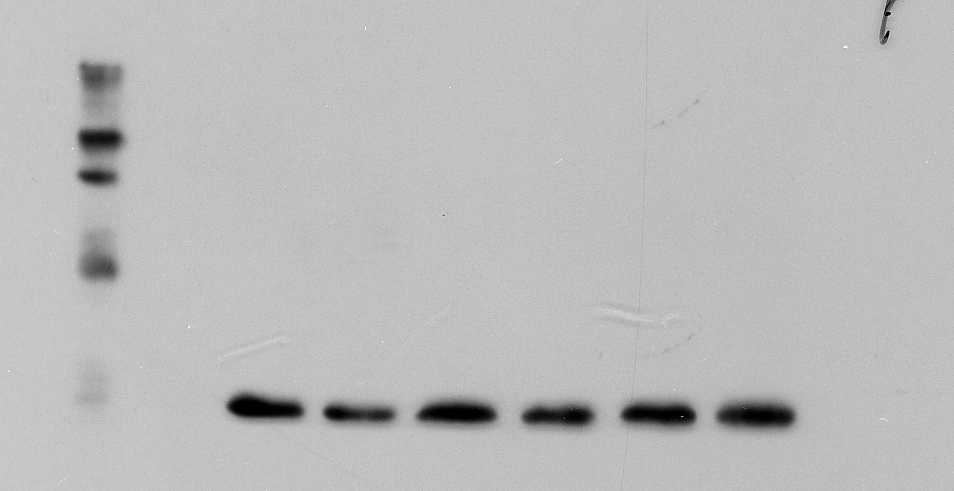


20kDa

4E-BP1


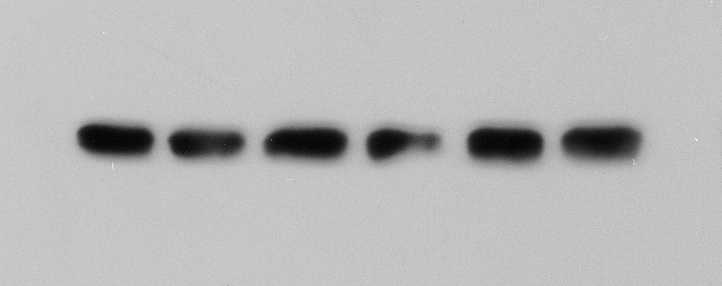


40kDa

β-actin


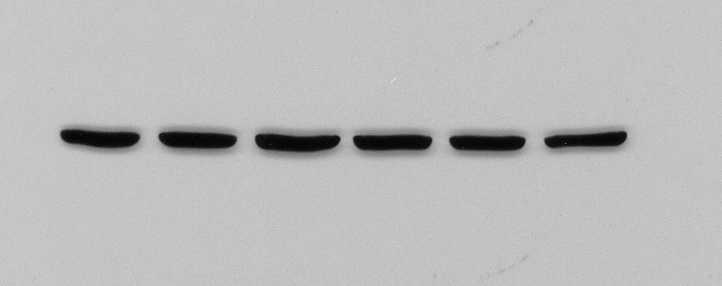


8h

20kDa

p-4E-BP1


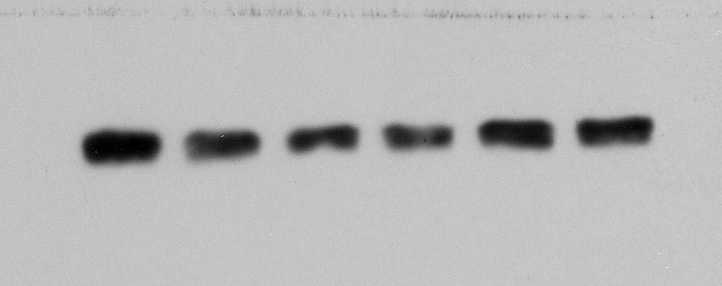


20kDa

4E-BP1


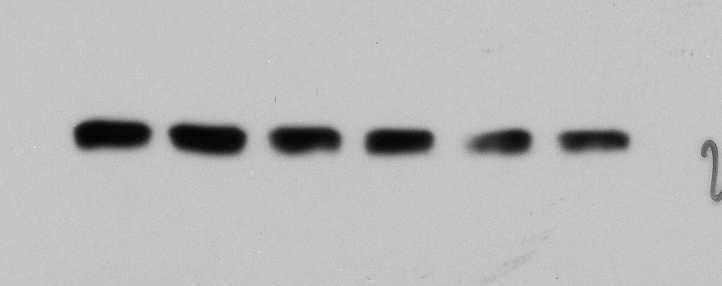


40kDa

β-actin


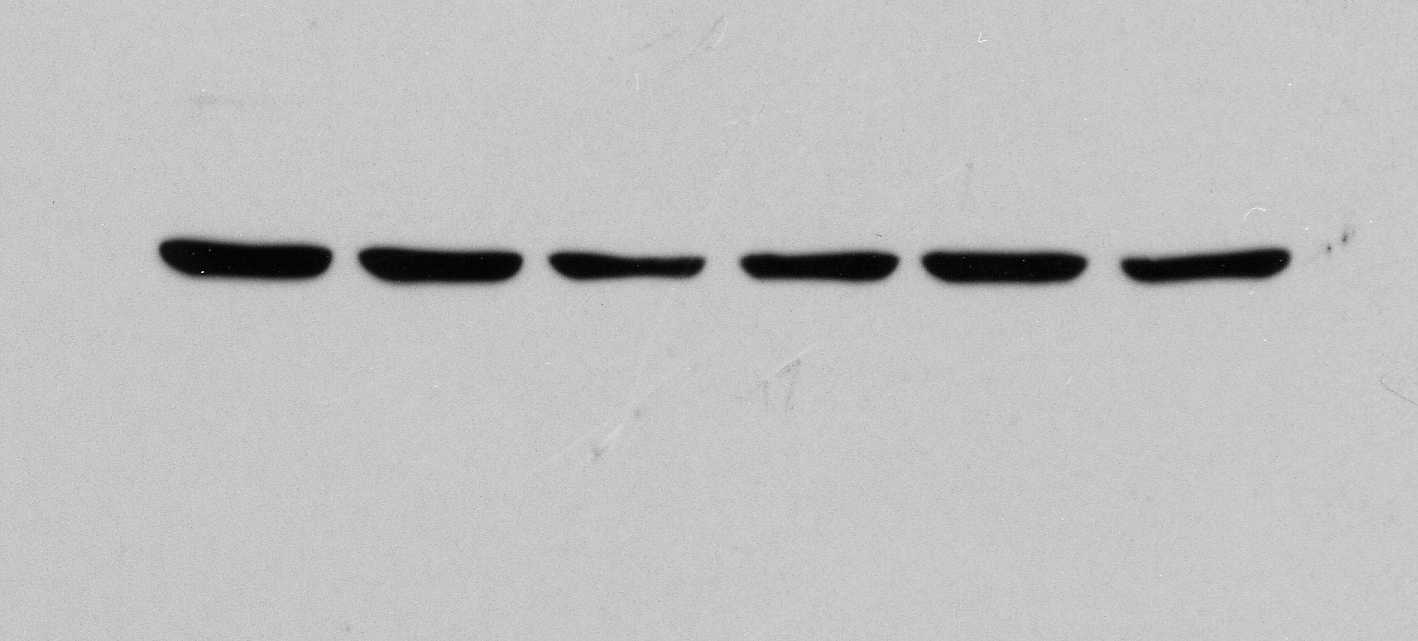


Extended Data Fig 7B

60kDa

p-AMPK


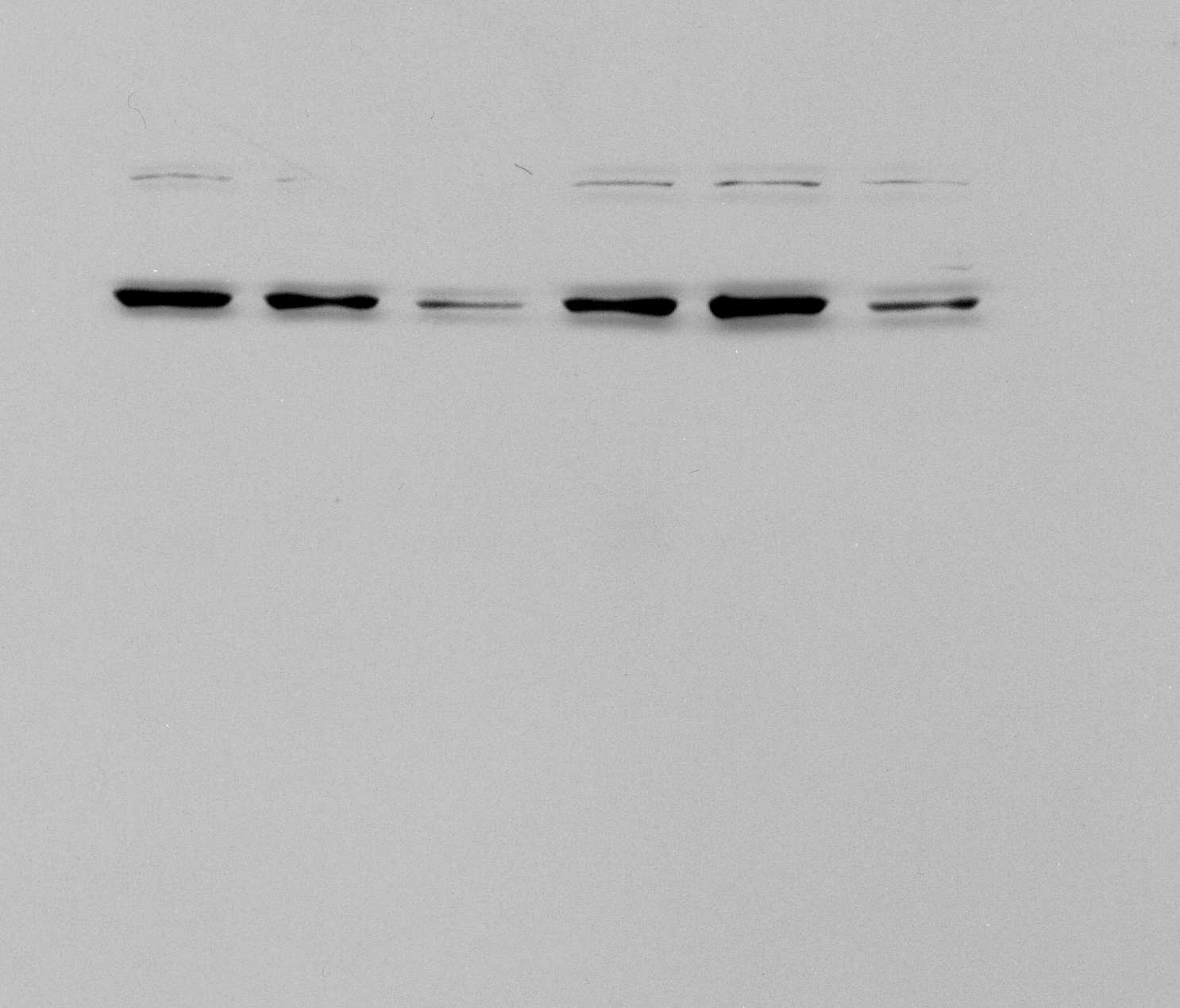


60kDa

AMPK


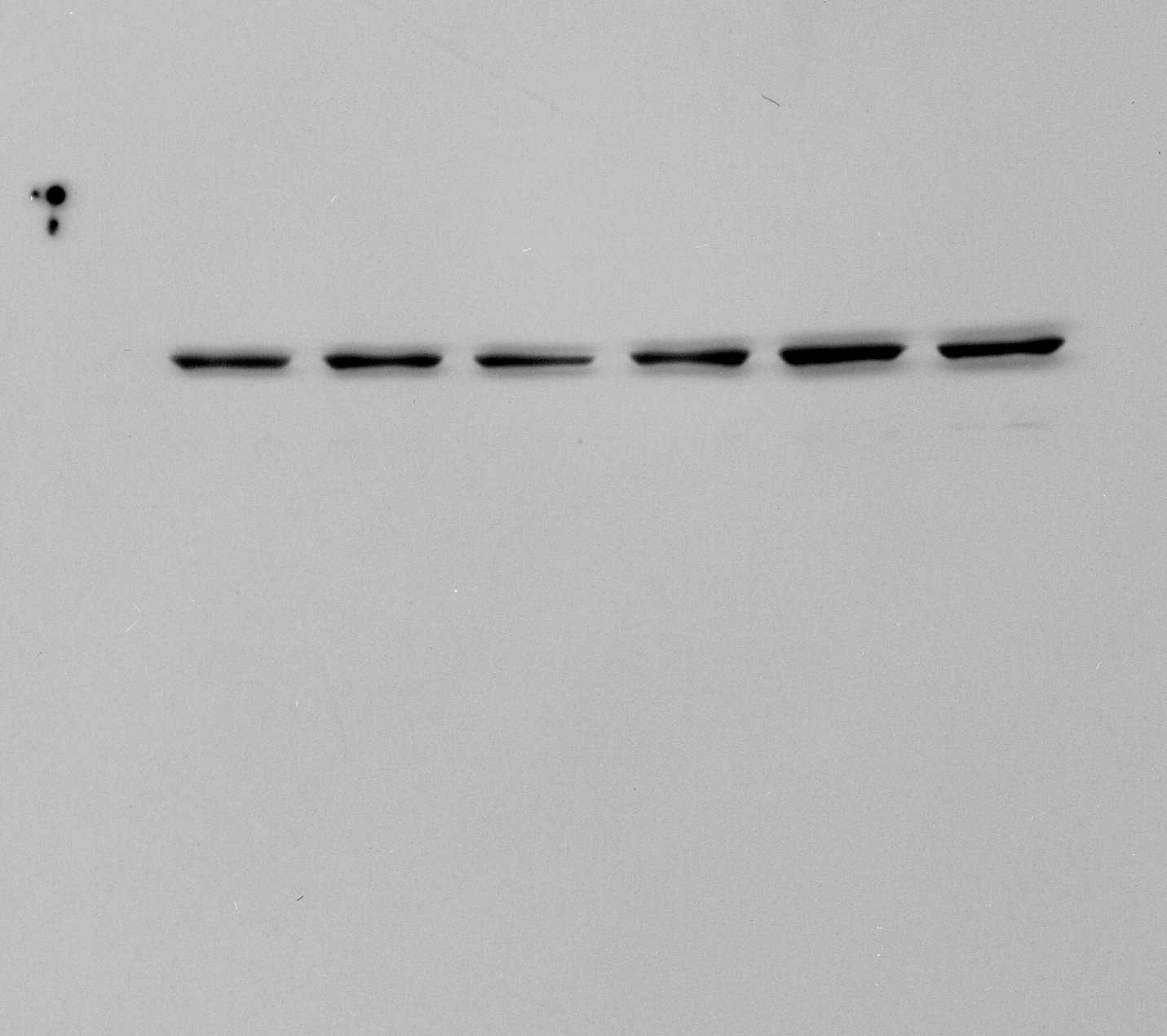


40kDa

β-actin


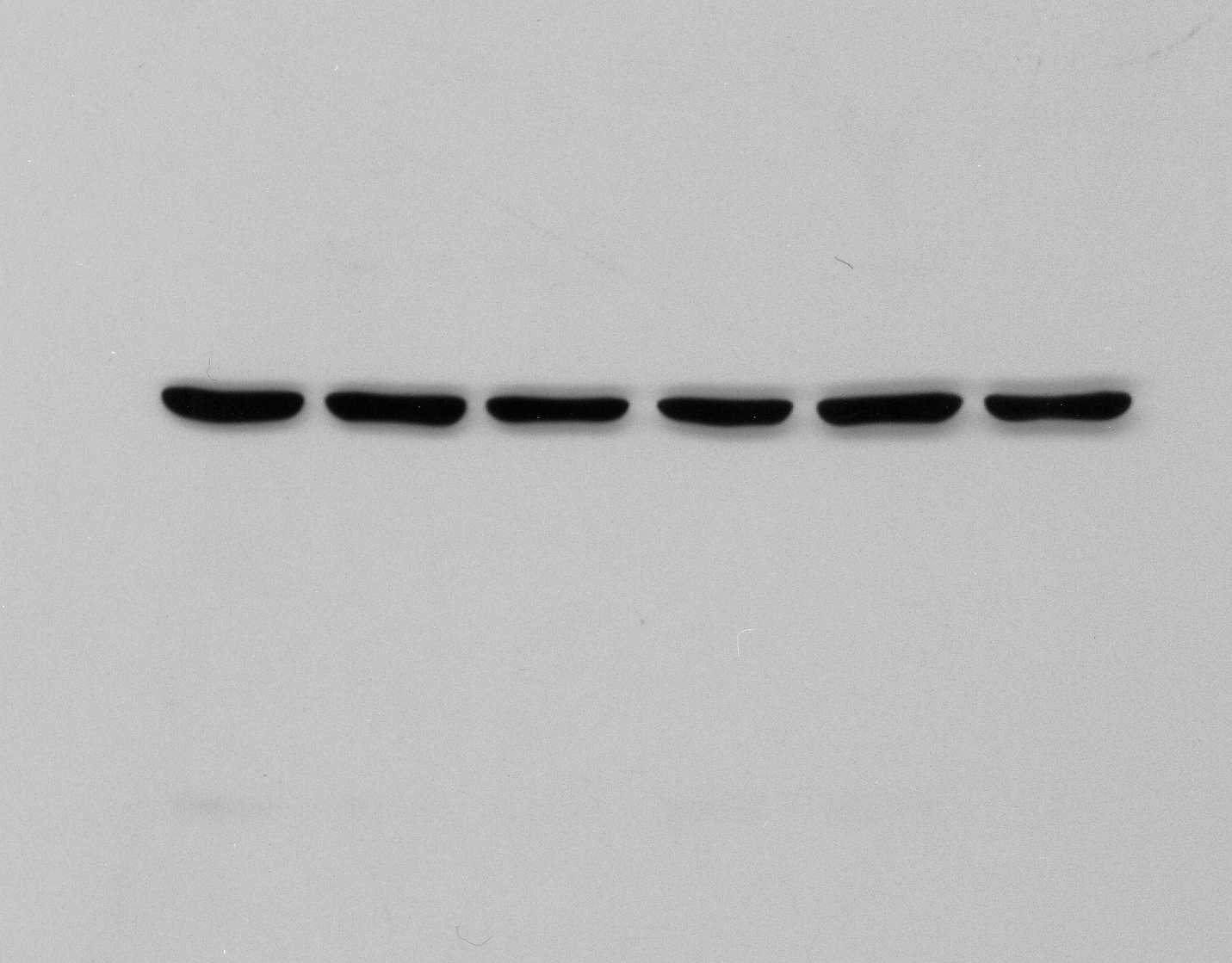

Supplement: S2 Fig — Uncropped images of western blots are displayed as shown. (DOCX) [file pone.0179586.s002.docx]
